# Prevalence of ‘*Candidatus* Accumulibacter phosphatis’ type II under phosphate limiting conditions

**DOI:** 10.1186/s13568-016-0214-z

**Published:** 2016-07-04

**Authors:** L. Welles, C. M. Lopez-Vazquez, C. M. Hooijmans, M. C. M. van Loosdrecht, D. Brdjanovic

**Affiliations:** Department of Environmental Engineering and Water Technology, UNESCO-IHE Institute for Water Education, Westvest 7, 2611AX Delft, The Netherlands; Department of Biotechnology, Delft University of Technology, Van der Maasweg 9, 2629 HZ Delft, The Netherlands; KWR watercycle research institute, Groningenhaven 7, Nieuwegein, The Netherlands

**Keywords:** Polyphosphate-accumulating organisms (PAO), Glycogen-accumulating organisms (GAO), Enhanced biological phosphate removal (EBPR), Polyphosphate content, Microbial ecology, Niche differentiation, ‘*Candidatus* Accumulibacter phosphatis’, ‘*Candidatus* Competibacter phosphatis’

## Abstract

P-limitation in enhanced biological phosphorus removal (EBPR) systems fed with acetate, has generally been considered as a condition leading to enrichment of organisms of the genotype’ *Candidatus* Competibacter phosphatis’ expressing the glycogen-accumulating organisms (GAO) phenotype. Recent studies have demonstrated in short-term experiments that organisms of the genotype ‘*Candidatus* Accumulibacter phosphatis’ clade I and II, known to express the polyphosphate-accumulating organisms (PAO) phenotype can switch to the GAO phenotype when poly-P is absent, but are performing the HAc-uptake at lower kinetic rates, where clade I showed the lowest rates. The objective of this study was to verify whether organisms of the genotype ‘*Candidatus* Accumulibacter phosphatis’ can also be enriched under P-limiting conditions while expressing a GAO phenotype and more specifically to see which specific clade prevails. A sequencing batch reactor was inoculated with activated sludge to enrich an EBPR culture for a cultivation period of 128 days (16 times the solids retention time) under P-limiting conditions. A mixed culture was obtained comprising of 49 % ‘*Candidatus* Accumulibacter phosphatis’ clade II and 46 % ‘*Candidatus* Competibacter phosphatis’. The culture performed a full GAO metabolism for anaerobic HAc-uptake, but was still able to switch to a PAO metabolism, taking up excessive amounts of phosphate during the aerobic phase when it became available in the influent. These findings show that P-limitation, often used as strategy for enrichment of ‘*Candidatus* Competibacter phosphatis’, does not always lead to enrichment of only ‘*Candidatus* Competibacter phosphatis’. Furthermore, it demonstrates that ‘*Candidatus* Accumulibacter phosphatis’ are able to proliferate in activated sludge systems for periods of up to 128 days or longer when the influent phosphate concentrations are just enough for assimilation purposes and no poly-P is formed. The ‘*Candidatus* Accumulibacter phosphatis’ retain the ability to switch to the PAO phenotype, taking up phosphate from the influent as soon as it becomes available.

**Electronic supplementary material:**

The online version of this article (doi:10.1186/s13568-016-0214-z) contains supplementary material, which is available to authorized users.

## Introduction

Enhanced biological phosphorus removal (EBPR) is a microbial process for removal of excessive amounts of phosphorus from wastewater through storage of intracellular polyphosphate (poly-P) by polyphosphate-accumulating organisms (PAO) and excess sludge wasting. Due to its high efficiency and cost-effectiveness, the process is widely implemented in biological wastewater treatment systems. When activated sludge is cycled through alternating anaerobic and aerobic zones, organisms with the PAO phenotype are able to take up phosphate from the liquid phase and store it as intracellular polyphosphate, leading to P-removal from the bulk liquid via PAO cell removal through the wastage of activated sludge. These organisms take up volatile fatty acids (VFA) under anaerobic conditions and store it as poly-β-hydroxyalkanoates (PHA) (Wentzel et al. 1985; Comeau et al. 1986; Mino et al. 1987). The uptake and storage of VFA requires energy, which can be generated by poly-P cleavage and subsequent release of ortho-phosphate. In the following aerobic phase, the organisms oxidize PHA and use the energy to restore their poly-P pool along with other metabolic processes. By linking microbial community composition with EBPR performance, “*Candidatus* Accumulibacter phosphatis” were identified as the organisms expressing the PAO phenotype in many laboratory EBPR systems (Bond et al. 1995, 1999; Hesselman et al. 1999; Crocetti et al. 2000) as well as full-scale waste water treatment plants (Zilles et al. 2002; Kong et al. 2004; Gu et al. 2005; He et al. 2005; Wong et al. 2005). Another group of organisms, ‘*Candidatus* Competibacter phosphatis’, are considered to compete with ‘*Candidatus* Accumulibacter phosphatis’ for acetate (HAc), expressing a so called glycogen-accumulating organisms (GAO) phenotype (Mino et al. 1987; Nielsen et al. 1999; Crocetti et al. 2002). The only difference is that for the GAO phenotype, glycogen is used as energy source instead of poly-P, for the uptake and storage of HAc and therefore they do not contribute to EBPR. Prevalence of ‘*Candidatus* Competibacter phosphatis’ is considered to be an important factor that leads to EBPR deterioration. Hence, the competition between ‘*Candidatus* Accumulibacter phosphatis’ and ‘*Candidatus* Competibacter phosphatis’ communities in EBPR processes has been the subject of several laboratory studies, often using highly enriched EBPR cultures expressing the PAO and GAO phenotypes (Oehmen et al. 2007).

To study factors affecting the competition between organisms that express the PAO and GAO phenotypes, it is necessary to obtain highly enriched ‘*Candidatus* Accumulibacter phosphatis’ and ‘*Candidatus* Competibacter phosphatis’ cultures. Influent phosphorus limitation has often been used as a strategy to enrich ‘*Candidatus* Competibacter phosphatis’ (Sudiana et al. 1999; Filipe et al. 2001; Zeng et al. 2003; Lopez-Vazquez et al. 2007). Mostly, since it was considered that ‘*Candidatus* Accumulibacter phosphatis’ were dependent on poly-P and would wash-out of the system once the phosphate concentrations became limiting. However, recent studies have shown in short-term experiments that ‘*Candidatus* Accumulibacter phosphatis’ are capable of performing a GAO metabolism (i.e. using glycogen to produce all of the required energy for VFA uptake) (Zhou et al. 2008; Acevedo et al. 2012) at lower kinetic rates. These new insights led to the speculation that P-limitation may not always lead to highly enriched ‘*Candidatus* Competibacter phosphatis’ cultures. This would also imply that biomass cultures enriched in previous studies, that seemed to be ‘*Candidatus* Competibacter phosphatis’ cultures, may have actually been ‘*Candidatus* Accumulibacter phosphatis’ cultures that performed a GAO metabolism, if no microbial characterization methods were applied that could distinguish ‘*Candidatus* Accumulibacter phosphatis’ and ‘*Candidatus* Competibacter phosphatis’ populations (as in Liu et al. 1997; Sudiana et al. 1999; Schuler and Jenkins 2003). Therefore, it is important to (re-)investigate which microbial populations will develop in EBPR systems operated under phosphate limiting conditions.

Besides the selection of ‘*Candidatus* Accumulibacter phosphatis’ and ‘*Candidatus* Competibacter phosphatis’, it is also important to verify which ‘*Candidatus* Accumulibacter phosphatis’ clades may proliferate under phosphate limiting conditions, as this may help to develop an enrichment strategy specifically for ‘*Candidatus* Accumulibacter phosphatis’ clade I or II. The intrinsic differences within the metabolic characteristics of the ‘*Candidatus* Accumulibacter phosphatis’ clades (Carvalho et al. 2007; Flowers et al. 2009; Slater et al. 2010), urges the need to study these metabolic differences and develop clade specific metabolic models for better understanding and description of EBPR processes. For this purpose, it is necessary to develop strategies for the selection of specific ‘*Candidatus* Accumulibacter phosphatis’ clades to obtain EBPR cultures highly enriched with specific clades.

A recent study suggested that ‘*Candidatus* Accumulibacter phosphatis’ clade II may have a competitive advantage over ‘*Candidatus* Accumulibacter phosphatis’ clade I under short-term poly-P depleted conditions (Acevedo et al. 2012). Moreover, through short-term batch tests it was shown that the HAc-uptake rate of ‘*Candidatus* Accumulibacter phosphatis’ clade II was four times faster than that of ‘*Candidatus* Accumulibacter phosphatis’ clade I under poly-P depleted conditions (Welles et al. 2015). Therefore, low influent P/C ratios or the periodical phosphate limitation may lead to ‘*Candidatus* Accumulibacter phosphatis’ clade II enrichments while high P/C influent ratios may support the development of ‘*Candidatus* Accumulibacter phosphatis’ clade I cultures.

The objectives of this study were: (i) to reinvestigate which bacterial populations can develop in EBPR systems inoculated with activated sludge operated for a long-term period under phosphate limiting conditions, (ii) to assess whether ‘*Candidatus* Accumulibacter phosphatis’ can get enriched and, if so, which specific clade would prevail, and (iii) to verify whether ‘*Candidatus* Accumulibacter phosphatis’ enriched under phosphate limiting conditions are capable of taking up excessive amounts of phosphate when they are suddenly exposed to high phosphate concentrations. This study contributes to the fundamental understanding of the ‘*Candidatus* Accumulibacter phosphatis’ metabolism and clade differentiation, helps to improve the strategies for ‘*Candidatus* Competibacter phosphatis’ and/or ‘*Candidatus* Accumulibacter phosphatis’ clade II enrichment and provides insight into the EBPR performance of WWTPs that may (periodically) suffer from phosphate limiting conditions.

## Materials and methods

### Bacterial enrichment under phosphate limiting conditions

#### Operation of sequencing batch reactors

An EBPR culture was enriched in a double-jacketed laboratory sequencing batch reactors (SBR) under the same conditions as those previously described for GAO by Welles et al. (2014). The SBR had a working volume of 2.5 l. Activated sludge from a municipal wastewater treatment plant (Hoek van Holland, The Netherlands) was used as inoculum. The SBR was operated in cycles of 6 h (2.25 h anaerobic, 2.25 h aerobic and 1.5 h settling phase) following similar operating conditions used in previous studies (Smolders et al. 1994). The SBR was operated at a pH value of 7.0 ± 0.05 and a temperature of 20 ± 1 °C. The applied solids retention time (SRT) was 8 days.

#### Synthetic medium

The SBR was fed with synthetic medium. The influent phosphate concentration was 0.071 P-mmol l^−1^ (2.2 mg PO_4_-P l^−1^) (Liu et al. 1997) and the acetate concentration was 12.6 C-mmol l^−1^ (373 mg HAc l^−1^, 400 mg COD l^−1^, 860 mg NaAc·3H_2_O), leading to an influent P/C ratio of 0.0056 (P-mol C-mol^−1^). Further details regarding other macronutrients and trace elements can be found in Smolders et al. (1994).

#### Monitoring of SBR

The performance of the SBR was regularly monitored by measuring the total suspended solids (TSS), volatile suspended solids (VSS) and inorganic suspended solids (ISS). The (pseudo) steady-state conditions in the reactor was confirmed by daily determination of the aforementioned parameters as well as online pH and dissolved oxygen (DO) profiles. When no significant changes of these parameters and the characteristic online DO and pH profiles were observed for a time interval of three SRT, the enrichment culture was considered to be in pseudo steady-state.

### Characterization of the microbial community

An estimation of the degree of enrichment of the bacterial populations of interest (‘Candidatus Accumulibacter phosphatis’ clade I, clade II and ‘Candidatus Competibacter phosphatis’) was undertaken via fluorescence in situ hybridization (FISH) microscopy analyses. Biomass samples were fixed in 4 % paraformaldehyde and incubated for 120 min at room temperature. After fixation, samples were centrifuged for 2 min at 6000 rpm, washed twice in 1× Phosphate buffer saline (PBS), re-suspended in an ethanol/PBS buffer mixture (volume 1:1) and finally stored at −20 °C. For hybridization, the fixed samples were dried on gelatin coated wells of hybridization slides. The samples were dehydrated by passing the microscope slides through 50, 80 and 100 % ethanol solutions for 3 min in each solution. After dehydration, the hybridization solution (10 µl) and 1 µL of oligonucleotide probe (tagged with the fluorescent labels Fluos, Cy5 or Cy3) solution (with concentration of 8.3, 5 and 5 pmol/L for Fluos, CY5 and CY3 labels, respectively) was added to each well, and the samples were immediately incubated for 2 h in a humid chamber at 46 °C. The hybridization buffer consisted of a mixture of 360 µl of 5 M NaCl, 40 µl of 1 M Tris (pH 8), 10 µL of a 10 % (w/v) sodium dodecylsulfate buffer (SDS), 700 µl of formamide, and 900 µl of MilliQ water (Amann et al. 1990; Crocetti et al. 2000, 2002; Daims et al. 1999). After hybridization, the microscope slides were washed at 48 °C for 12 min by immersing them into 50 ml of washing solution. The washing solution consisted of 800 µl of 5 M NaCl, 500 µl of 0.5 M EDTA, 1000 µl of 1 M Tris (pH8), and 50 µl of 10 % SDS(w/v). The samples were dried and prepared with 2 ml antifade fluorescent mounting oil. The probes used to target the organisms of interest are shown in Table 1. The whole bacterial community was targeted by the EUB338mix (mix of general bacteria probes EUB, EUB II and EUB III). ‘Candidatus Accumulibacter phosphatis’ was targeted by the PAO651 probe whereas GAOMIX probe (mixture of probes GAOQ431 and GAOQ989) was used to target ‘Candidatus Competibacter phosphatis’. ‘Candidatus Accumulibacter phosphatis’ clade I (clade IA and other type I clades) and ‘Candidatus Accumulibacter phosphatis’ clade II (clade IIA, IIC and IID) were targeted by the probes Acc-1-444 and Acc-2-444, respectively. Hybridized samples were examined with Zeiss Axioplan-2 epifluorescence microscope. The quantification of the ‘Candidatus Accumulibacter phosphatis’ and ‘Candidatus Competibacter phosphatis’ biomass fractions (of the entire bacterial community) and the ‘Candidatus Accumulibacter phosphatis’ clade I and II fractions (of the ‘Candidatus Accumulibacter phosphatis’ community) in the biomass was carried out via FISH image analysis using the free ImageJ software package (version 1.47b, Wayne Rasband, National Institute of Health, USA). For each quantification 18 randomly selected FISH images were analysed. For the PAO and GAO fractions, the surface area of cells binding the specific PAO651 and GAOmix probes were expressed as the mean percentage of the surface area of cells binding the entire bacterial community (EUBmix). For the PAO I and PAO II fraction, the surface area of cells binding the specific PAOmix and GAOmix probes were expressed as the mean percentage of the surface area of cells binding the entire PAO community (PAOmix). The standard error of the mean (SEM) was calculated as the standard deviation of the area percentage divided by the square root of the number of images for each quantification.

**Table 1:** Overview of probes used to target the organisms of interest for the FISH microscopic analysis of the enrichment culture

Reference	Probe	Sequence (5′–3′)	Target	Formamide (%)
Amann et al. (1990)	EUB	GCTGCCTCCCGTAGGAGT	Many but not all Bacteria	0–70
Daims et al. (1999)	EUB II	GCAGCCACCCGTAGGTGT	Planctomycetales	0–50
Daims et al. (1999)	EUB III	GCTGCCACCCGTAGGTGT	Verrucomicrobiales	0–50
Crocetti et al. (2000)	PAO 651	CCCTCTGCCAAACTCCAG	‘Candidatus Accumulibacter phosphatis’	35
Crocetti et al. (2002)	GAOQ431	TCCCCGCCTAAAGGGCTT	‘Candidatus Competibacter phosphatis’	35
Crocetti et al. (2002)	GAOQ989	TTCCCCGGATGTCAAGGC	‘Candidatus Competibacter phosphatis’	35
Flowers et al. (2009)	Acc-I-444	CCCAAGCAATTTCTTCCCC	‘Candidatus Accumulibacter phosphatis’ Clade IA and other Type I clades	35
Flowers et al. (2009)	Acc-II-444	CCCGTGCAATTTCTTCCCC	‘Candidatus Accumulibacter phosphatis’ Clade IIA, IIC and IID	35

### Characterization of anaerobic carbon and phosphate conversions

When the SBR reached pseudo steady-state conditions, a cycle was intensively monitored to determine the biomass kinetic rates and stoichiometry of the anaerobic conversions. In addition to the above described parameters, orthophosphate (PO_4_^3−^-P), acetate (HAc-C), PHA and glycogen were measured in the cycle. To verify if the enriched culture was able to take up excessive amounts of phosphate, two consecutive cycles were conducted in which a concentrated phosphate solution was added at the end of the anaerobic phase prior to the aerobic stage. This phosphate addition led to an increase of 0.65 P-mmol l^−1^ in the reactor. In the following aerobic phase, the phosphate concentration was intensively monitored.

### Analyses

The determinations of TSS, VSS and ISS were performed in triplicates in accordance with Standard Methods (A.P.H.A. 1995). For the TSS analysis, well-mixed samples with a volume of 10 mL were filtered through weighed standard glass-fiber filters (Whatman GF/C, diameter 47 mm). After filtration of the samples, an additional 10 ml of tap water was filtered to wash away dissolved solids in the remaining liquid phase in the filters and residues. The residues retained on the filters were dried for 24 h at 103–105 °C. The increase in weight of the filters represented the total suspended solids (TSS) concentration. The residues were ignited for 3 h at 520 °C. The remaining solids represented the fixed suspended solids (ISS) concentrations, while the weight lost on ignition represented the volatile suspended solids (VSS) concentrations. PO4-P was determined by the ascorbic acid method in accordance with Standard Methods (A.P.H.A. 1995). HAc was determined using a Varian 430-GC Gas Chromatograph (GC), equipped with a split injector (split ratio 1:10), a WCOT Fused Silica column with a FFAP-CB coating (25 m × 0.53 mm × 1 μm), and coupled to a FID detector. Helium gas was used as carrier gas. The temperature of the injector, column and detector was 200, 105 and 300 °C, respectively. Glycogen was determined according to the method described by Smolders et al. (1994) but with an extended digestion of 5 h in 5 ml 0.9 mol l^−1^ HCl, using 5 mg of freeze dried biomass as described by Lanham et al. (2012). The poly-β-hydroxybutyrate PHB and poly-β-hydroxyvalerate (PHV) contents of freeze dried biomass were determined according to the method described by Smolders et al. (1994). The non-soluble total phosphorus concentration (Pns) at the end of the aerobic (and beginning of the cycle was determined on the basis of the steady state mass balance for phosphorus using Eqs. 1a–d. Pns at the end of the anaerobic phase was determined by subtracting the ortho-phosphate concentration at the end of the anaerobic phase from the steady state aerobic Pns.

1a $$\begin{aligned}& {\text{d }}\left( {{\text{T}}_{{{\text{P}},{\text{e}}}} *{\text{V}}_{\text{p}} } \right)/{\text{dt }} \\ & \quad= {\text{ Q}}_{\text{i}} * \, \left( {{\text{T}}_{{{\text{P}},{\text{i}}}} - {\text{ T}}_{{{\text{P}},{\text{e}}}} } \right) \, - {\text{ Q}}_{\text{w}} *{\text{ TSS }}*{\text{ f}}_{{{\text{P}},{\text{TSS}}}} = \, 0 \end{aligned}$$

1b $$\begin{aligned} & {\text{Q}}_{\text{i}} /{\text{V}}_{\text{p}} * \, \left( {{\text{T}}_{{{\text{P}},{\text{i}}}} - {\text{ T}}_{{{\text{P}},{\text{e}}}} } \right) \, - {\text{ Q}}_{\text{w}} /{\text{ V}}_{\text{p}} *{\text{ TSS }}*{\text{ f}}_{{{\text{P}},{\text{TSS}}}} \\ & \quad = 0 \end{aligned}$$

1c $$\begin{aligned}& 1/{\text{HRT }}* \, \left( {{\text{T}}_{{{\text{P}},{\text{i}}}} - {\text{ T}}_{{{\text{P}},{\text{e}}}} } \right) \, - { 1}/{\text{SRT }}*{\text{ TSS }}*{\text{ f}}_{{{\text{P}},{\text{TSS}}}} \\ & \quad= 0 \end{aligned}$$

1d $${\text{Pns}} = {\text{ SRT}}/{\text{HRT }}* \, \left( {{\text{T}}_{{{\text{P}},{\text{i}}}} - {\text{ T}}_{{{\text{P}},{\text{e}}}} } \right)$$

where TSS: concentration of total suspended solids, Pns: concentration of non-soluble total phosphorus, T_P,i_: total phosphorus concentration in the influent, T_P,e_: total phosphorus concentration in the effluent, f_P,TSS_: ratio of total P per TSS, V_p_: working volume of reactor, Q_i_: influent flow rate, Q_w_: wastage of activated sludge flow rate, HRT: hydraulic retention time and SRT: solids retention time.

### Determination of kinetic and stoichiometric parameters

The kinetic rate of interest was the anaerobic HAc-uptake rate. This rate was expressed as maximum active biomass specific rate based on the HAc profiles observed in the tests as described by Smolders et al. (1994) and Brdjanovic et al. (1997). The stoichiometric ratios of interest were P/HAc, PHV/PHB, PHV/HAc, PHB/HAc and gly/HAc.

## Results

### Enrichment of EBPR culture under phosphate limiting conditions

**Fig. 1:**
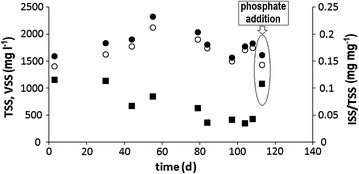
Solids concentrations in the SBR-reactor during long-term operation: total suspended solids (TSS) concentration (*filled circle*), volatile suspended solids (VSS) concentration (*open* *circle*) and the ratio of inorganic suspended solids (ISS)/TSS (*filled* *square*)

After inoculation, the online pH and DO profiles indicated that within approximately 10 SRT (80 days) the performance of the reactor reached steady-state conditions. In Fig. 1, the TSS and VSS concentrations as well as the ISS/TSS ratio are shown. Although the TSS and VSS concentrations fluctuated during the period of enrichment, the average TSS and VSS concentrations (1.9 gTSS l^−1^, 1.8 gVSS l^−1^) of this period were in the range of concentrations observed in similar previous studies. The ISS/TSS ratio of the inoculum was 0.12 mg mg^−1^. This gradually decreased to a value of 0.04 mg mg^−1^, except for the last data point which was obtained after conducting a batch experiment with an additional phosphate feed. The low ISS/TSS ratio observed in this study under steady-state conditions (0.04 mg mg^−1^), in comparison to typical ISS/TSS ratios of high grade PAO enrichments [0.60 mg mg^−1^ (Schuler and Jenkins 2003a)] indicates that the sludge did not contain significant quantities of poly-P as expected from the experimental design with the low influent phosphate concentration. On the basis of the steady state mass balance (and the low influent phosphate concentration), the Pns (non-soluble total phosphorus concentration) in the system was determined as 1.1 P-mmol l^−1^ (35 mgP/l) (Eq. 1d) and the Pns/VSS ratio was determined as 0.019 mgP mgVSS-1. These values are in the range of normal non-EBPR biomass (0.023 Metcalf and Eddy 2003) and demonstrate that there is practically no poly-P present in the sludge. As ‘*Candidatus* Accumulibacter phosphatis’ are considered to rely on intracellular poly-P for anaerobic substrate uptake, it was expected that they were no longer present in this sludge.

**Fig. 2:**
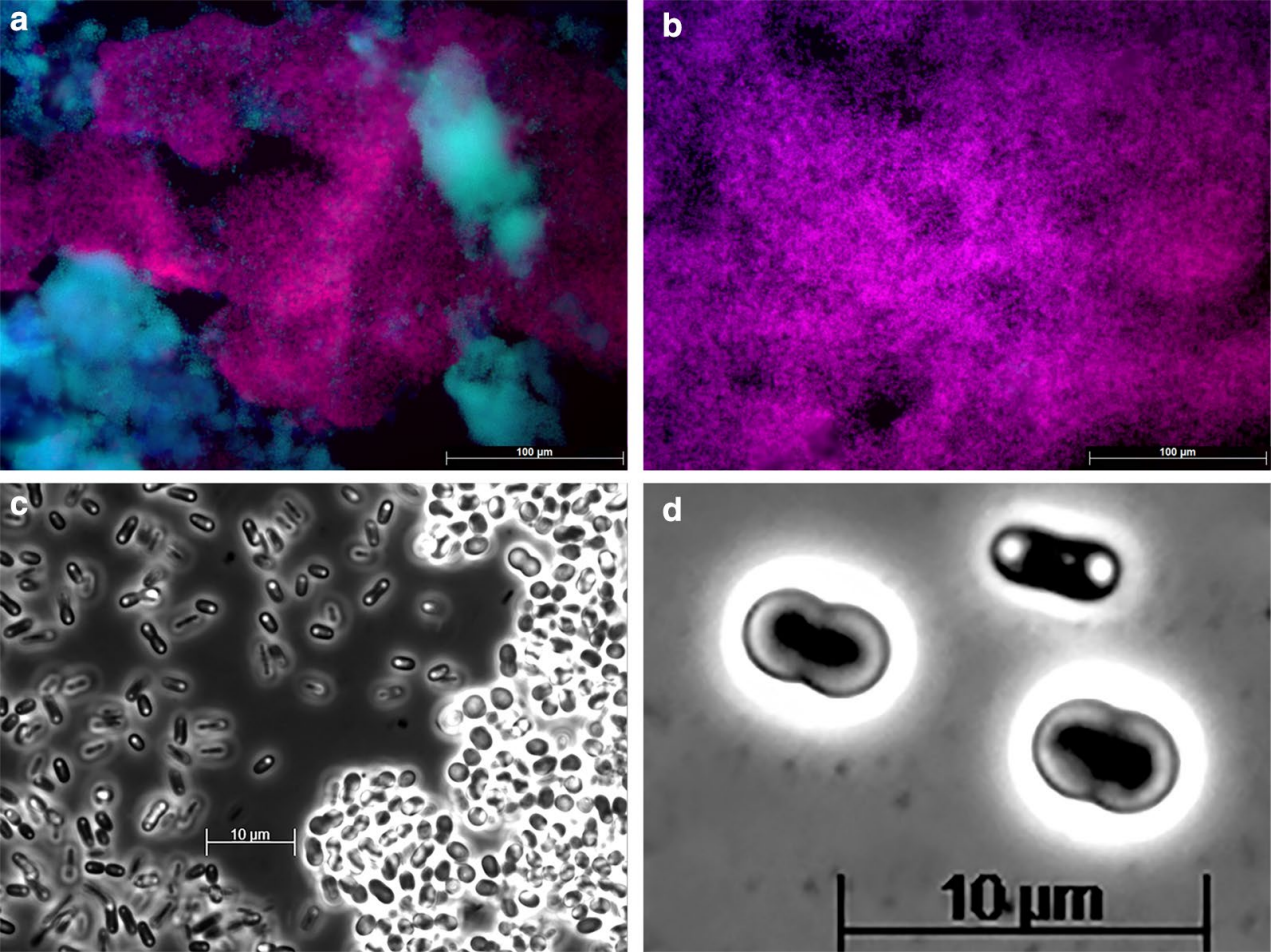
Representative FISH-microscopy images (**a**, **b**) and phase contrast images (**c**, **d**) showing the distribution of bacterial populations in the enriched biomass culture. In **a**, *blue* (EUB mix, Cy5): all bacteria other than ‘*Candidatus* Accumulibacter phosphatis’ and ‘*Candidatus* Competibacter phosphatis’; *purple* (superposition of PAO mix 651, Cy3 and EUB mix, Cy5): ‘*Candidatus* Accumulibacter phosphatis’; and *cyan green* (superposition of GAO mix, Fluos and EUB mix, Cy5): ‘*Candidatus* Competibacter phosphatis’. In **b**, *blue* (PAO mix 651, Cy5): all potential PAO that do not stain with specific probes for ‘*Candidatus* Accumulibacter phosphatis’ type I and II, *purple* (superposition of PAO mix 651, Cy5 and Acc II, Cy3): ‘*Candidatus* Accumulibacter phosphatis’ type II, and *cyan* *green* (superposition of PAO mix 651, Cy5 and Acc I, Fluos): ‘*Candidatus* Accumulibacter phosphatis’ type I. In **c**,* dark cells*: bacteria with the typical ‘*Candidatus* Accumulibacter phosphatis’ morphology;* bright cells*: bacteria with the typical ‘*Candidatus* Competibacter phosphatis’ morphology. In **d**,* small size*: bacteria with the typical ‘*Candidatus* Accumulibacter phosphatis’ morphology;* big size*: bacteria with the typical ‘*Candidatus* Competibacter phosphatis’ morphology

After approximately 14 SRT (113 days), the composition of the microbial community was analyzed. In Fig. 2, FISH images and phase contrast images of the enriched culture are shown. Figure 2a remarkably shows that both ‘*Candidatus* Accumulibacter phosphatis’ and ‘*Candidatus* Competibacter phosphatis’ were present in similar quantities. Additional FISH analyses showed that among the ‘*Candidatus* Accumulibacter phosphatis’ clades, clade II was dominant whereas the presence of clade I was negligible. FISH quantification showed that the ‘*Candidatus* Accumulibacter phosphatis’ and ‘*Candidatus* Competibacter phosphatis’ fractions of the total bacterial population were 49 ± 6 and 46 ± 7 %, respectively (see Additional file 1 for raw FISH images). Based on these ‘*Candidatus* Accumulibacter phosphatis’ and ‘*Candidatus* Competibacter phosphatis’ fractions, the ‘*Candidatus* Accumulibacter phosphatis’/‘*Candidatus* Competibacter phosphatis’ ratio was around 1:1. The ‘*Candidatus* Accumulibacter phosphatis’ clade I and II fractions with regard to the ‘*Candidatus* Accumulibacter phosphatis’ population were 0 ± 0 % (n = 16) and 99 ± 2 % (n = 16), respectively. In Fig. 2c and d, phase contrast images of the biomass are presented, showing the prevalence (Fig. 2c) of two groups of organisms with distinct morphotypes (coccus and rod) (Fig. 2d). By comparing the phase contrast and the FISH images (Additional file 1), the organisms with the rod morphology were identified as ‘*Candidatus* Accumulibacter phosphatis’ and those with the coccus morphology as ‘*Candidatus* Competibacter phosphatis’.

### Biochemical conversions

**Fig. 3:**
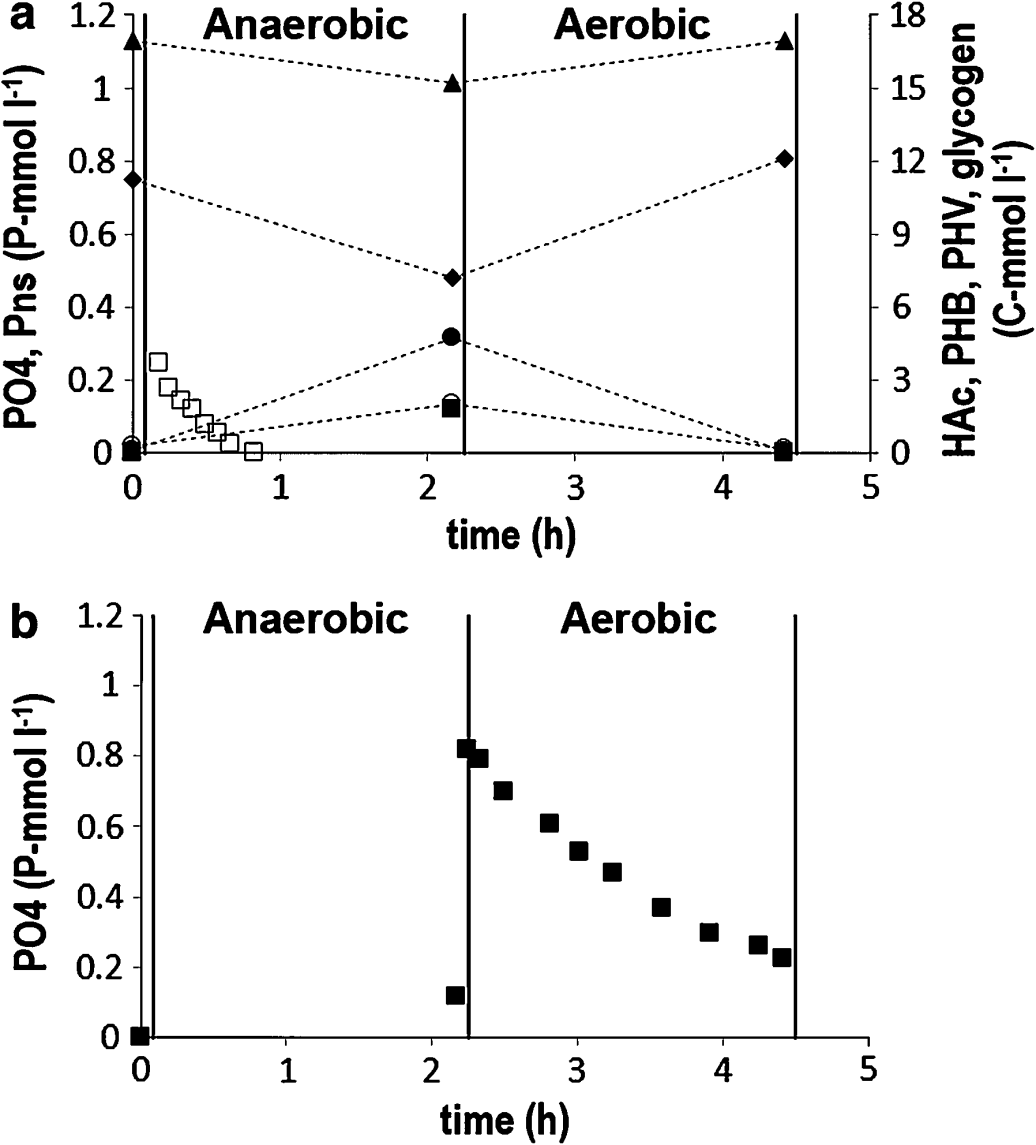
Biochemical conversion observed during a steady state cycle (**a**) and after the addition of phosphate in the end of the anaerobic phase (**b**). Dissolved components (without* connecting lines*): Acetate (Hac, *open square*) and ortho-phosphate (PO4, *filled square*). Suspended components (with *connecting lines*): Non-soluble total phosphorus (Pns, *filled triangle*), poly-β-hydroxybutyrate (PHB, *filled circle*), poly-β-hydroxyvalerate (PHV, *open circle*) and glycogen (*filled* *diamond*)

To confirm the absence of a poly-P dependent metabolism for anaerobic substrate uptake under steady-state conditions, the carbon and phosphate conversions were monitored during one cycle after approximately 11 SRT (84 days) (Fig. 3a). During the anaerobic phase, all HAc (6.25 C-mmol l^−1^) was taken up in less than 1 h with an active biomass specific HAc-uptake rate of 144 (C-mmol (C-mol h)^−1^) and this HAc-uptake was coupled to a net P-release of 0.09 P-mmol l^−1^ (2.7 mgP l^−1^). This led to a P-release/HAc-uptake ratio of 0.01 P-mol C-mol^−1^, which indicates a negligible involvement of poly-P in the anaerobic conversions when compared to typical ratios [P-release/HAc-uptake ratios as high as 0.75 P-mol C-mol^−1^ (Schuler and Jenkins 2003)] of high grade PAO enrichment cultures. The stoichiometric values of the anaerobic carbon and phosphate conversions are given in Table 2. The high gly/HAc ratio, high PHV/PHB as well as the other stoichiometric values are characteristic for a fully glycogen dependent metabolism as observed in enriched GAO cultures, in which the energy production from poly-P degradation in the PAO metabolism is substituted by energy production through glycogen conversion into PHA.

**Table 2:** Comparison of stoichiometric and kinetic values of the anaerobic conversions determined in this study and previous studies with enriched PAO and GAO cultures

References	Sequencing batch reactor (SBR) Batch reactor (BR),	Organisms PAO II, PAO I, PAO, GAO	SRT (days)	HRT (h)	pH	Influent [Ca^2+^] (mg l^−1^)	VSS/ TSS (mg mg^−1^)	PHV/PHB (C-mol C-mol^−1^)	PHV/HAc (C-mol C-mol^−1^)	PHB/HAc (C-mol C-mol^−1^)	P/HAc (P-mol C-mol^−1^)	Gly/HAc (C-mol C-mol^−1^)	${\text{q}}_{{{\text{SA}},{\text{ana}}}}^{\text{MAX}}$ C-mol (C-mol h)^−1^
This study	SBR	PAO II and GAO	8	12	7 ± 0.1	3.8	0.96	0.37	0.54	1.45	0.03	1.28	0.14
Zeng et al. (2003)	SBR	GAO	6.6	8	7 ± 0.1	6.8	0.97	0.38	0.52	1.39	NA	1.20	0.16–0.18
Lopez-Vazquez et al. (2007)	SBR	GAO	10	12	7 ± 0.1	3.8	0.9	0.34	0.69	1.28	0.01	1.20	0.20
Welles et al. (2015)	SBR	PAO II	8	12	7 ± 0.05	3.8	0.75	0.19	0.23	1.24	0.22	0.96	0.15
	BR	PAO II	NA	NA	7 ± 0.1	3.8	0.93	0.27	0.32	1.19	0.01	0.98	0.08
	SBR	PAO I	8	12	7 ± 0.1	3.8	0.58	0.07	0.09	1.27	0.64	0.29	0.18
	BR	PAO I	NA	NA	7 ± 0.1	3.8	0.95	0.33	0.37	1.09	0.02	1.28	0.02
Zhou et al. (2008)	SBR	PAO	8	24	7.0–8.0	1.3	0.6	0.06	0.07	1.18	0.62	0.46	NA
	BR	PAO	NA	NA	7.5 ± 0.01	1.3	NA	0.37	0.46	1.24	0.06	1.03	0.07
Acevedo et al. (2012)	SBR	PAO I	8	12	7.0–8.9	10	0.45	0.04	0.05	1.31	0.7	0.38	NA
	SBR	PAO I, II	8	12	7.0–8.9	10	0.92	0.16	0.28	1.74	0.08	1.08	NA
Tian et al. (2013)	SBR	PAO I	16	12	7 ± 0.1	3.8	NA	0.1	0.13	1.31	0.56	0.55	NA
Welles et al. 2014	SBR	GAO	8	12	7.0	3.8	0.97	NA	NA	NA	0.012	1.2	0.15
Filipe et al. (2001)	SBR	?^b^	7	12	6.8–7.1	3.8	NA	0.31	0.38	1.26	NA	0.83	0.24
^Sudiana et al.^ (1999) Reactor AL^a^	SBR	?^c^	NA	NA	6.8–7.2	NA	NA	0.24	0.4	1.7	0.02	1.30	0.06–0.08
^Liu et al.^ (1997) ^a^	SBR	?	8	6	7 ± 0.1	7.6	NA	NA	NA	NA	0.02	1.37	0.04
^Schuler and Jenkins^ (2003) ^a^	SBR	?	4	12	7.15–7.25	16	NA	NA	NA	NA	0.11	1.19	0.03

${\text{q}}_{{{\text{SA}},{\text{ana}}}}^{\text{MAX}}$ specific maximum anaerobic HAc-uptake rate

*NA* not applicable

^a^Calculated assuming that the VSS fully comprised of active biomass

^b^DGGE banding patterns indicated that 75 % of the population belonged to γ-proteobacteria, while with FISH analysis, using specific probes developed to target the dominant γ-proteobacteria in the DGGE banding pattern, showed that 35 % of the population stained positive for these γ-proteobacteria

^c^FISH analysis revealed that β-proteobacteria were dominant, comprising about one-third of the sludge

To verify if the ‘*Candidatus* Accumulibacter phosphatis’, that seemed to be present, were able to take up phosphate, two consecutive cycle tests were conducted after approximately 14 SRT (113 days), in which 0.65 P-mmol l^−1^ (20 mgP l^−1^) was added to the reactor at the end of each anaerobic phase (Fig. 3b). In the first cycle, 0.6 P-mmol l^−1^ (18 mgP l^−1^) was taken up while in the second cycle 0.7 P-mmol l^−1^ (21 mgP l^−1^) was taken up (data not shown) in total 1.26 P-mmol l^−1^ (39 mgP l^−1^) in two consecutive cycles, which is about 17 times more phosphorus removal in comparison to the phosphorus removal observed during the normal operation of the system. Considering that for the assimilation of biomass in the two consecutive cycles only 0.07 P-mmol l^−1^ (2.1 mgP l^−1^) was needed and that chemical precipitation at pH 7.0 with low calcium concentrations is not likely to occur, this ortho-phosphate removal confirms that the ‘*Candidatus* Accumulibacter phosphatis’ observed by microscopy were present, and that they were able to remove excessive amounts of phosphate from the liquid phase. This was further confirmed by the increase in the ISS/TSS ratio. The storage of 1.26 P-mmol l^−1^ (39 mgP l^−1^) as poly-P is equivalent to 0.13 gISS l^−1^, assuming a poly-P composition of (KMg(PO_3_)_3_). With a TSS concentration of 1.6 gTSS l^−1^, this increase in the ISS concentration would make a difference of 0.08 mg mg^−1^ in the ISS/TSS ratio, which approximately corresponds to the increase in the ISS/TSS ratio shown in Fig. 1.

## Discussion

### Enrichment of mixed cultures of ‘*Candidatus* Accumulibacter phosphatis’ and ‘*Candidatus* Competibacter phosphatis’

Bacterial populations cultivated in continuous and sequencing batch reactors will always wash-out (i) from continuously operated reactors if the maximum growth rate of the organisms is lower than the dilution rate or (ii) in SBR reactors if the maximum net growth per cycle is lower than the net biomass removal per cycle. If, bacteria are not able to grow at all, then in the ideal case it would take only 1 SRT to wash-out more than 64 % of the bacterial population that was present originally and 3 SRT to wash-out at least 95 %. In this study a mixed culture of ‘*Candidatus* Accumulibacter phosphatis’ and ‘*Candidatus* Competibacter phosphatis’ was obtained after an enrichment period of 14 SRT under phosphate limiting conditions, which comprised 49 ± 6 % ‘*Candidatus* Accumulibacter phosphatis’ clade II and 46 ± 7 % ‘*Candidatus* Competibacter phosphatis’. Therefore, this study clearly demonstrated that ‘*Candidatus* Accumulibacter phosphatis’ clade II were able to proliferate under phosphate limiting conditions. In a previous study, the ‘*Candidatus* Accumulibacter phosphatis’ fractions were determined in six activated sludge treatment plants in the Netherlands (Lopez-Vazquez et al. 2008), ranging from 6 to 16 % (with an average of 9.2 %) of the total bacterial community, while the ‘*Candidatus* Competibacter phosphatis’ fractions ranged from 0.4 to 3.2 % (showing an average of 1.7 %). The considerably higher ‘*Candidatus* Accumulibacter phosphatis’ fraction (49 %) obtained in the enriched culture of this study suggests that with practically depleted poly-P reserves ‘*Candidatus* Accumulibacter phosphatis’ clade II were still able to compete with ordinary heterotrophic bacteria by taking up VFA in the anaerobic phase using a metabolism that is not dependent on poly-P. The stoichiometry of the anaerobic carbon and phosphate conversions confirmed that the mixed population made full use of a ‘*Candidatus* Competibacter phosphatis’ metabolism under anaerobic conditions (Table 2). Additional experiments confirmed that under aerobic conditions PAO were able to perform a ‘*Candidatus* Accumulibacter phosphatis’ metabolism when phosphate was added to the system (Fig. 3). These results are in line with previous findings regarding a ‘*Candidatus* Competibacter phosphatis’ enrichment study performed by Lopez-Vazquez et al. (2007), in which a mixed culture was obtained containing 75 % ‘*Candidatus* Competibacter phosphatis’ and 20 % ‘*Candidatus* Accumulibacter phosphatis’ after an enrichment period of 10 SRT. In two previous ‘*Candidatus* Competibacter phosphatis’ enrichment trials performed by the authors of the present study, a reactor was inoculated with sludge from a highly enriched ‘*Candidatus* Accumulibacter phosphatis’ clade II reactor (Welles et al. 2015) assuming that the sludge contained ‘*Candidatus* Competibacter phosphatis’ since most of HAc was taken up through a GAO metabolism. In addition, it was expected that any PAO present would rapidly be washed out of the system due to the low influent P concentration (assuming that they were fully dependent on poly-P) leading to a highly enriched ‘*Candidatus* Competibacter phosphatis’ culture. However, in those enrichment trials FISH analyses revealed that (i) the sludge used as inoculum was rich in ‘*Candidatus* Accumulibacter phosphatis’ II and did not contain ‘*Candidatus* Competibacter phosphatis’ (Welles et al. 2015) and (ii) that under phosphate limiting conditions (like those applied in the present study) ‘*Candidatus* Accumulibacter phosphatis’ II were still dominant after 15 and 16 SRT of operation (data not shown). In spite of the presumably low poly-P contents, those cultures were able to completely remove HAc during the anaerobic phase.

### Competition between ‘*Candidatus* Accumulibacter phosphatis’ clade I, clade II and ‘*Candidatus* Competibacter phosphatis’

Enrichment of the specific ‘*Candidatus* Accumulibacter phosphatis’ clade II is in agreement with the findings of Welles et al. (2015a) where clade II showed HAc uptake rates four times higher than those of clade I under poly-P depleted conditions. In those studies, it was suggested that clade II has a competitive advantage over clade I under phosphate limiting conditions. In a study performed by, Tian et al. (2013) an enriched ‘*Candidatus* Accumulibacter phosphatis’ clade I culture was not able to complete the HAc-uptake during the anaerobic phase (36 days SRT, 0.5 days HRT, at 10 °C, pH 7.0 with an influent HAc concentration of 12.5 C-mmol l^−1^ (375 mg l^−1^) and an anaerobic phase of 2.25 h) when the phosphate concentration was limiting 0.071 P-mmol l^−1^ (2.2 mgP l^−1^ influent ortho-phosphate concentration, resulting in an influent P/C ratio of 0.0056 P-mol C-mol^−1^). Similar to this observation, Schuler and Jenkins (2003) also observed a leakage of HAc to the aerobic phase (4 days SRT, 0.5 days HRT, 20 °C, pH 7.0, 6.26 C-mmol l^−1^ (188 mg l^−1^) influent HAc concentration and 1.83 h anaerobic phase duration) when phosphate became limiting (at an influent P/C ratio lower than 0.019 P-mol C-mol^−1^). Based on the stoichiometry and kinetic rates reported, it seemed that the culture of Schuler and Jenkins (2003a) was also a ‘*Candidatus* Accumulibacter phosphatis’ clade I dominated culture (Welles et al. 2015). The findings in this study, in the study of Tian et al. (2013) and Schuler and Jenkins (2003a) support the hypothesis that under P-limiting conditions, ‘*Candidatus* Accumulibacter phosphatis’ clade II can proliferate in the system by adjusting its metabolism to low P/C influent ratios while clade I cannot. At high influent P/C ratios (above 0.04 P-mol C-mol^−1^) with a high poly-P content, clade I may exhibit faster HAc-uptake rates and outcompete clade II.

In the study of Welles et al. (2015) the active biomass specific HAc-uptake rates of PAO (80 C-mmol (C-mol h)^−1^) determined in short-term batch tests under poly-P depleted conditions seemed to be significantly lower than that of ‘*Candidatus* Competibacter phosphatis’ (150–200 C-mmol (C-mol h^−1^) (Zeng et al. 2003; Lopez-Vazquez et al. 2007; Welles et al. 2014), suggesting that ‘*Candidatus* Competibacter phosphatis’ would still be able to outcompete ‘*Candidatus* Accumulibacter phosphatis’ after a few SRT. However, the ‘*Candidatus* Accumulibacter phosphatis’ fractions observed even after enrichment periods of 14–16 SRT were still very significant. This indicates that a population shift from ‘*Candidatus* Accumulibacter phosphatis’ clade II to ‘*Candidatus* Competibacter phosphatis’ needed a long period due to a high initial ‘*Candidatus* Accumulibacter phosphatis’ clade II/‘*Candidatus* Competibacter phosphatis’ ratio and a relatively small difference in the HAc-uptake rates of the ‘*Candidatus* Accumulibacter phosphatis’ and ‘*Candidatus* Accumulibacter phosphatis’ enriched in this system. Alternatively, ‘*Candidatus* Competibacter phosphatis’ cultivated under certain controlled operational conditions in laboratory reactors only reach through adaptation their typical maximum activity after a certain period of cultivation and are not directly competitive from the moment of inoculation.

### Implications

Limitation of phosphorus has often been used as a strategy to enrich ‘*Candidatus* Competibacter phosphatis’ (Sudiana et al. 1999; Filipe et al. 2001; Zeng et al. 2003; Lopez-Vazquez et al. 2007). This study has demonstrated that it is an unreliable selection strategy that does not always lead to highly enriched ‘*Candidatus* Competibacter phosphatis’ cultures. In addition, the carbon and phosphorus conversions cannot be used as reliable indicators to assess the presence of ‘*Candidatus* Competibacter phosphatis’. Therefore, to conduct a microbial characterization, using FISH or other microbial identification techniques is always recommended in EBPR studies. Results of previous studies performed on organisms that express the GAO phenotype, without any microbial identification (e.g. Sudiana et al. 1999; Liu et al. 1997; Schuler and Jenkins 2003b), may therefore be questioned, especially if the enrichment period was limited to a few SRT only. The observation that both ‘*Candidatus* Accumulibacter phosphatis’ and ‘*Candidatus* Competibacter phosphatis’ are able to perform a GAO metabolism but at different rates helps to explain the broad range of HAc-uptake rates reported from enriched ‘GAO’ cultures. In the studies of Sudiana et al. (1999), Liu et al. (1997) and Schuler and Jenkins (2003b) where the presence of ‘*Candidatus* Competibacter phosphatis’ was not reported, HAc-uptake rates range from 0.04 to 0.08 C-mol (C-mol biomass h)^−1^, while the HAc-uptake rates in studies where the presence of ‘*Candidatus* Competibacter phosphatis’ was confirmed vary between 0.16 and 0.20 C-mol (C-mol biomass h)^−1^ (Lopez-Vazquez et al. 2007; Zeng et al. 2003; Filipe et al. 2001; Welles et al. 2014). Possibly under P-limited conditions lower HAc uptake rates could be associated to ‘*Candidatus* Accumulibacter phosphatis’ clade II enrichments and higher rates to ‘*Candidatus* Competibacter phosphatis’ enrichments.

This study also suggests that the ‘*Candidatus* Accumulibacter phosphatis’ clade II/‘*Candidatus* Competibacter phosphatis’ fractions in the inoculum have a significant impact on the time that is needed for enrichment of ‘*Candidatus* Competibacter phosphatis’ cultures and that a long enrichment period may be needed to obtain highly enriched cultures. Welles et al. (2014) were able to obtain a highly enriched ‘*Candidatus* Competibacter phosphatis’ culture after 44 SRT (352 days) under the same operational conditions, although minor traces of ‘*Candidatus* Accumulibacter phosphatis’ clade II were still present in the biomass. The selection of ‘*Candidatus* Competibacter phosphatis’ may be accelerated when the operating temperature of the reactor is increased (Lopez-Vazquez et al. 2007, 2008), but on the other hand this could lead to selection of specific ‘*Candidatus* Competibacter phosphatis’ that normally prevail in processes at elevated temperature and therefore those ‘*Candidatus* Competibacter phosphatis’ cultures may not be representative in context of the research on wastewater treatment in moderate climate conditions. Further research is needed to define appropriate strategies to enrich ‘*Candidatus* Accumulibacter phosphatis’ clade I, clade II and ‘*Candidatus* Competibacter phosphatis’ for their further study and characterization.

The findings drawn from this study also indicate that temporary limitation of ortho-phosphate by temporal overdosing of iron in activated sludge systems or fluctuations in the influent P/C ratio of industrial wastewater may not have deleterious effects under similar operational conditions (pH = 7.0 and T = 20 °C) on the ability of the activated sludge to perform EBPR once the ortho-phosphate levels in the influent are restored. Past studies have indicated that simultaneous chemical precipitation and enhanced biological phosphorus removal in activated sludge systems, led to a decreased biological phosphorus removal activity and accompanied storage of Poly-P during the periods of iron addition, due to a competition for ortho-phosphate by the chemical and biological mechanisms (De Haas et al. 2000, 2004). However, depletion of poly-P storage pools in ‘*Candidatus* Accumulibacter phosphatis’ clade II, would not severely affect its ability to proliferate in the system and therefore clade II has the potential to remain in the systems for several SRT while performing a GAO metabolism. Once the ortho-phosphate concentrations are restored, ‘*Candidatus* Accumulibacter phosphatis’ clade II may be still present in the system and perform EBPR activity instantly. Temporal overdosing of iron in activated sludge systems or fluctuations in the influent P/C ratio of industrial wastewaters may be problematic in activated sludge systems dominated by ‘*Candidatus* Accumulibacter phosphatis’ clade I but may not be problematic in sludge systems dominated by clade II. Based on the duration of this study and considering that the applied SRT in full-scale activated sludge plants achieving phosphorus and nitrogen removal usually vary between 8 and 30 days, ‘*Candidatus* Accumulibacter phosphatis’ clade II could be able to prevail in activated sludge systems for 80 up to 300 days under phosphate limiting conditions.

## Conclusions

A mixed culture of ‘*Candidatus* Accumulibacter phosphatis’ clade II and ‘*Candidatus* Competibacter phosphatis’ was enriched after a cultivation period of 14–16 SRT under ortho-phosphate limiting conditions. The ‘*Candidatus* Accumulibacter phosphatis’ and ‘*Candidatus* Competibacter phosphatis’ fractions of the total microbial community were around 49 ± 6 and 46 ± 7 %, respectively. In particular, all PAO were closely related to ‘*Candidatus* Accumulibacter phosphatis’ Clade II. Under anaerobic conditions, the mixed culture performed a typical GAO metabolism in which all energy for HAc-uptake was produced by the conversion of glycogen. However, under aerobic conditions ‘*Candidatus* Accumulibacter phosphatis’ were capable of taking up excessive amounts of phosphate when additional phosphate was added to the reactor. This study suggests that limitation of phosphate, often used as a strategy for the enrichment of ‘*Candidatus* Competibacter phosphatis’, does not always lead to high ‘*Candidatus* Competibacter phosphatis’ enrichment and that the carbon conversions often used as indicator for ‘*Candidatus* Competibacter phosphatis’ enrichments are no longer reliable as stand-alone indicators. Furthermore, the development of ‘*Candidatus* Accumulibacter phosphatis’ clade II suggests that clade II has a competitive advantage over clade I under phosphate limiting conditions. From a practical perspective, this study demonstrates that ‘*Candidatus* Accumulibacter phosphatis’ may be able to proliferate under phosphate limiting conditions in activated sludge systems for periods of up to 128 days (16 SRT) or longer while being able to take up phosphate aerobically as soon as it is available in the influent.

## Additional file

10.1186/s13568-016-0214-z FISH-microscopy images used for quantification.
