# Supplementary material for: Prevalence of ‘Candidatus Accumulibacter phosphatis’ type II under phosphate limiting conditions
Source: AMB Express. 2016 Jul 4;6:44. doi: 10.1186/s13568-016-0214-z (PMC4932009; doi:10.1186/s13568-016-0214-z)
Supplement: Supplementary file 1 — 10.1186/s13568-016-0214-z FISH-microscopy images used for quantification. [file 13568_2016_214_MOESM1_ESM.pptx]

## Slide 1
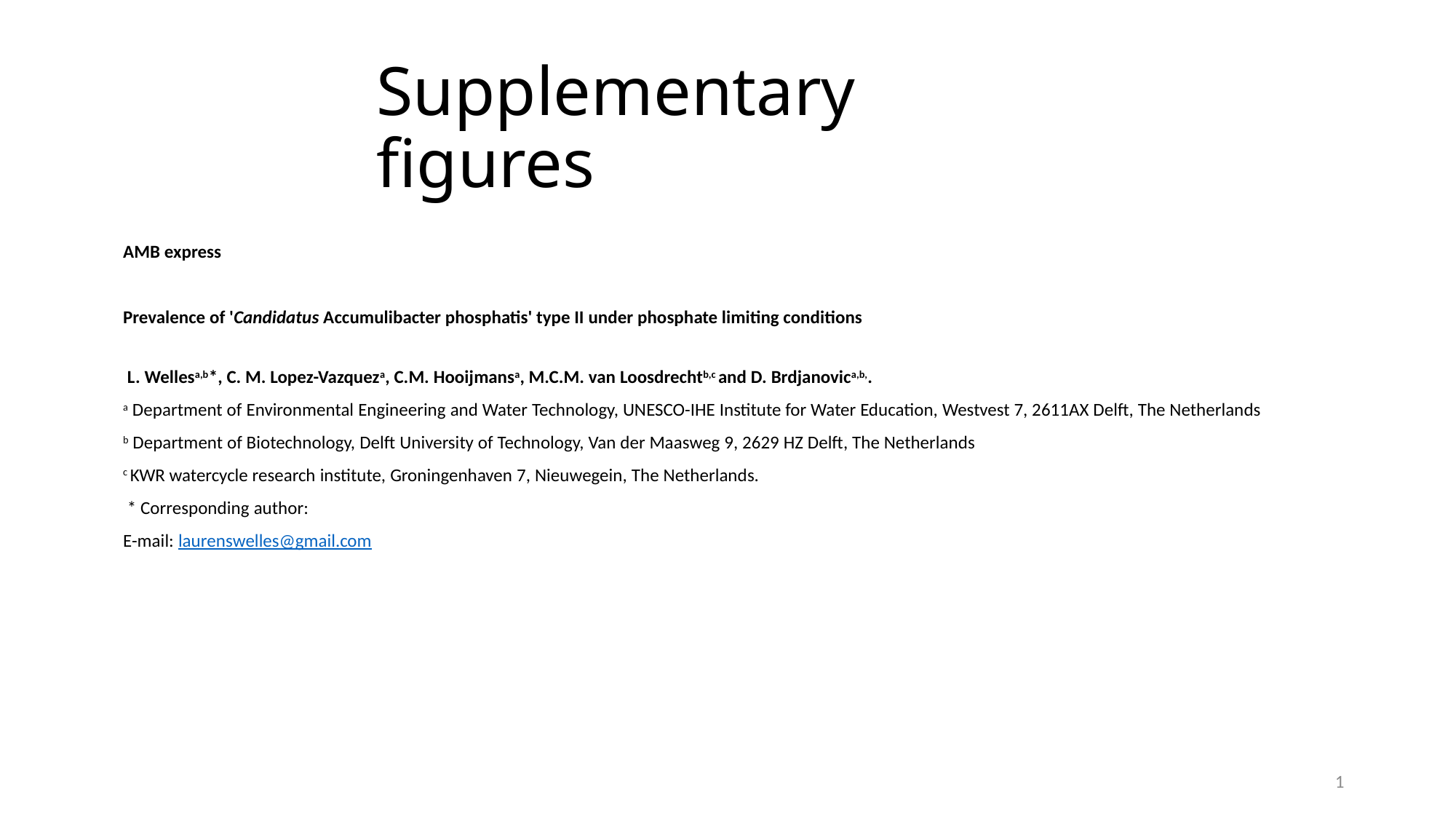

# Supplementary figures
AMB express
Prevalence of 'Candidatus Accumulibacter phosphatis' type II under phosphate limiting conditions
 L. Wellesa,b*, C. M. Lopez-Vazqueza, C.M. Hooijmansa, M.C.M. van Loosdrechtb,c and D. Brdjanovica,b,.
a Department of Environmental Engineering and Water Technology, UNESCO-IHE Institute for Water Education, Westvest 7, 2611AX Delft, The Netherlands
b Department of Biotechnology, Delft University of Technology, Van der Maasweg 9, 2629 HZ Delft, The Netherlands
c KWR watercycle research institute, Groningenhaven 7, Nieuwegein, The Netherlands.
 * Corresponding author:
E-mail: laurenswelles@gmail.com
1

## Slide 2
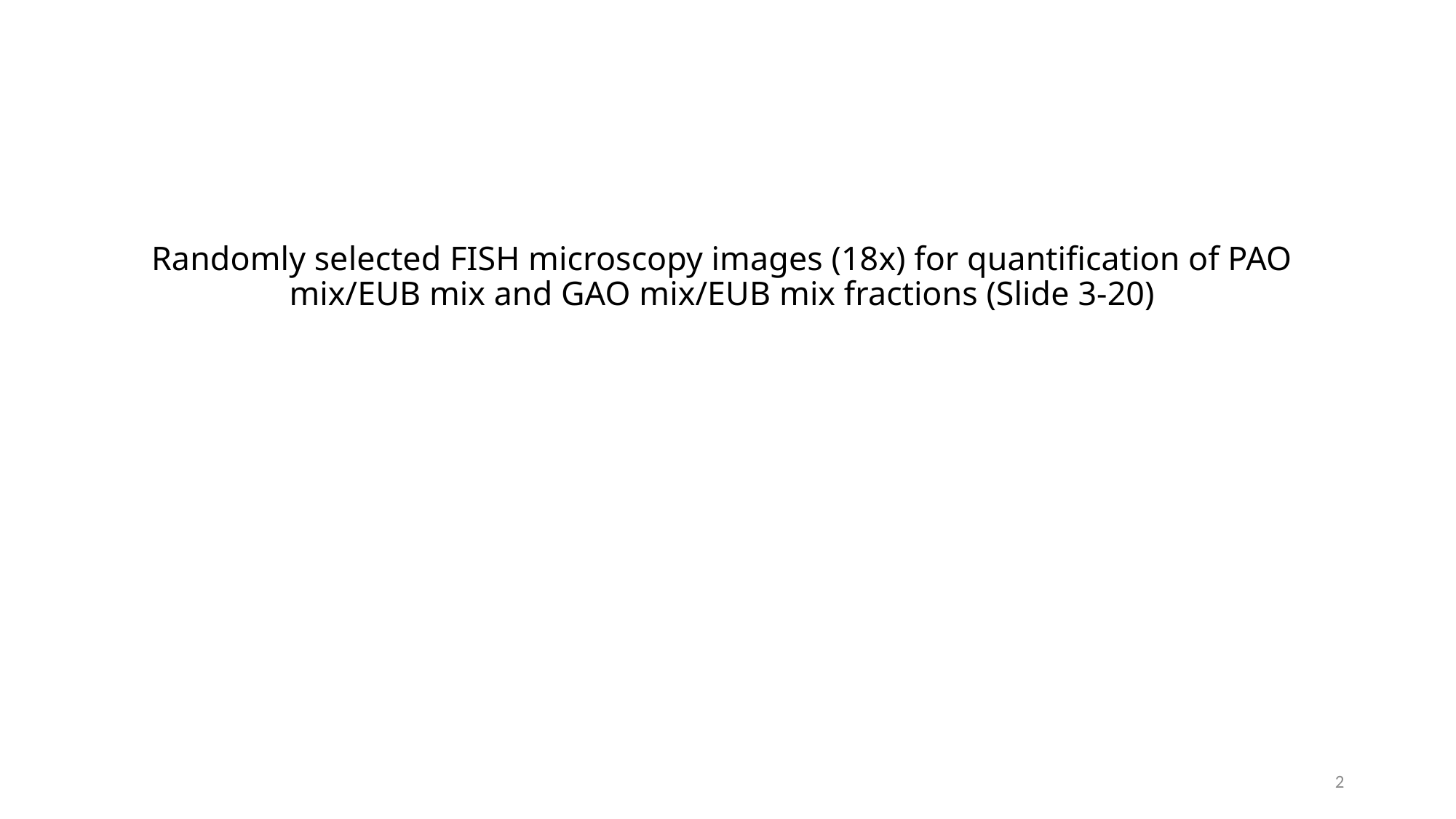

# Randomly selected FISH microscopy images (18x) for quantification of PAO mix/EUB mix and GAO mix/EUB mix fractions (Slide 3-20)
2

## Slide 3
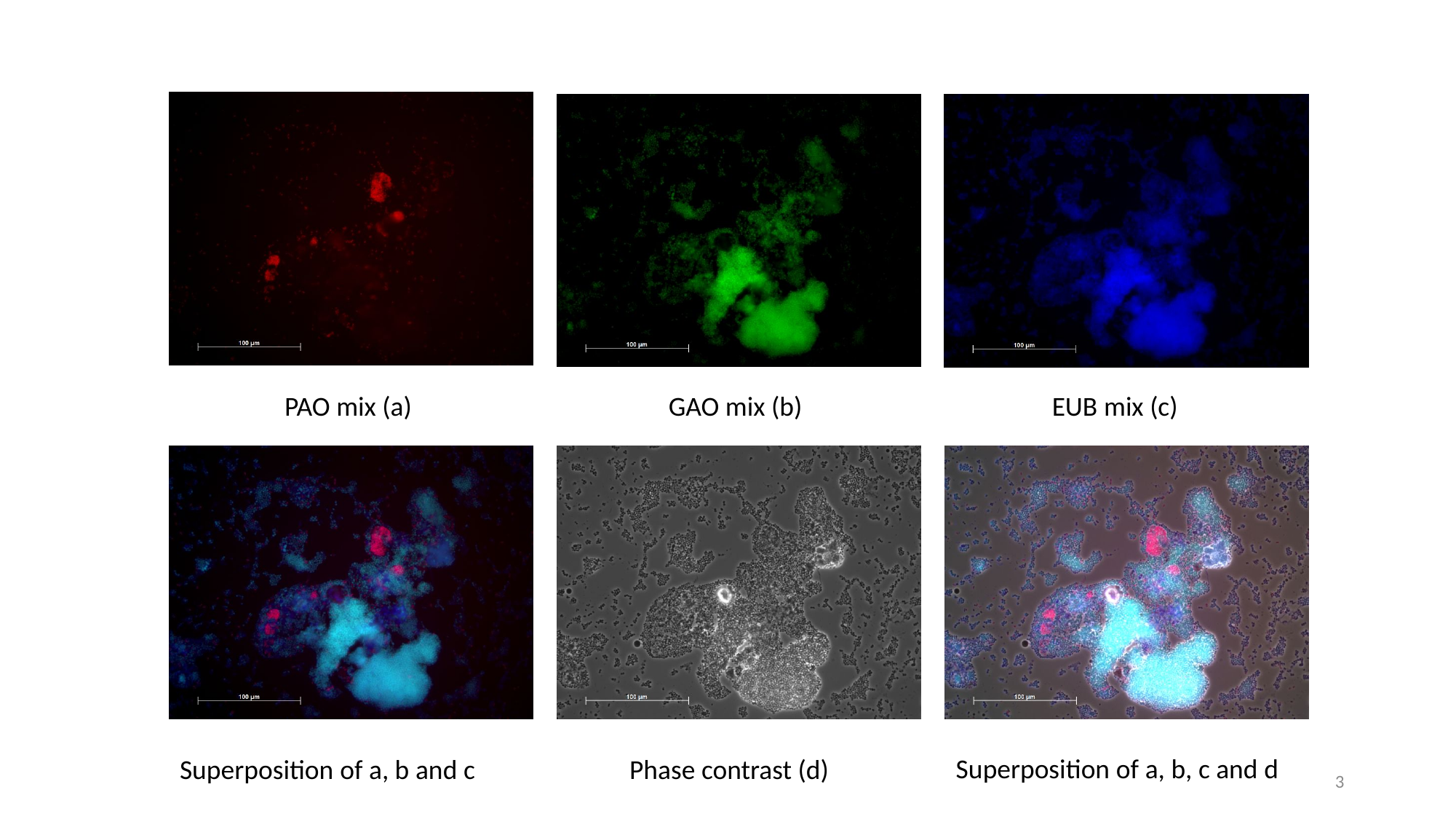

PAO mix (a)
GAO mix (b)
EUB mix (c)
Superposition of a, b, c and d
Superposition of a, b and c
Phase contrast (d)
3

## Slide 4
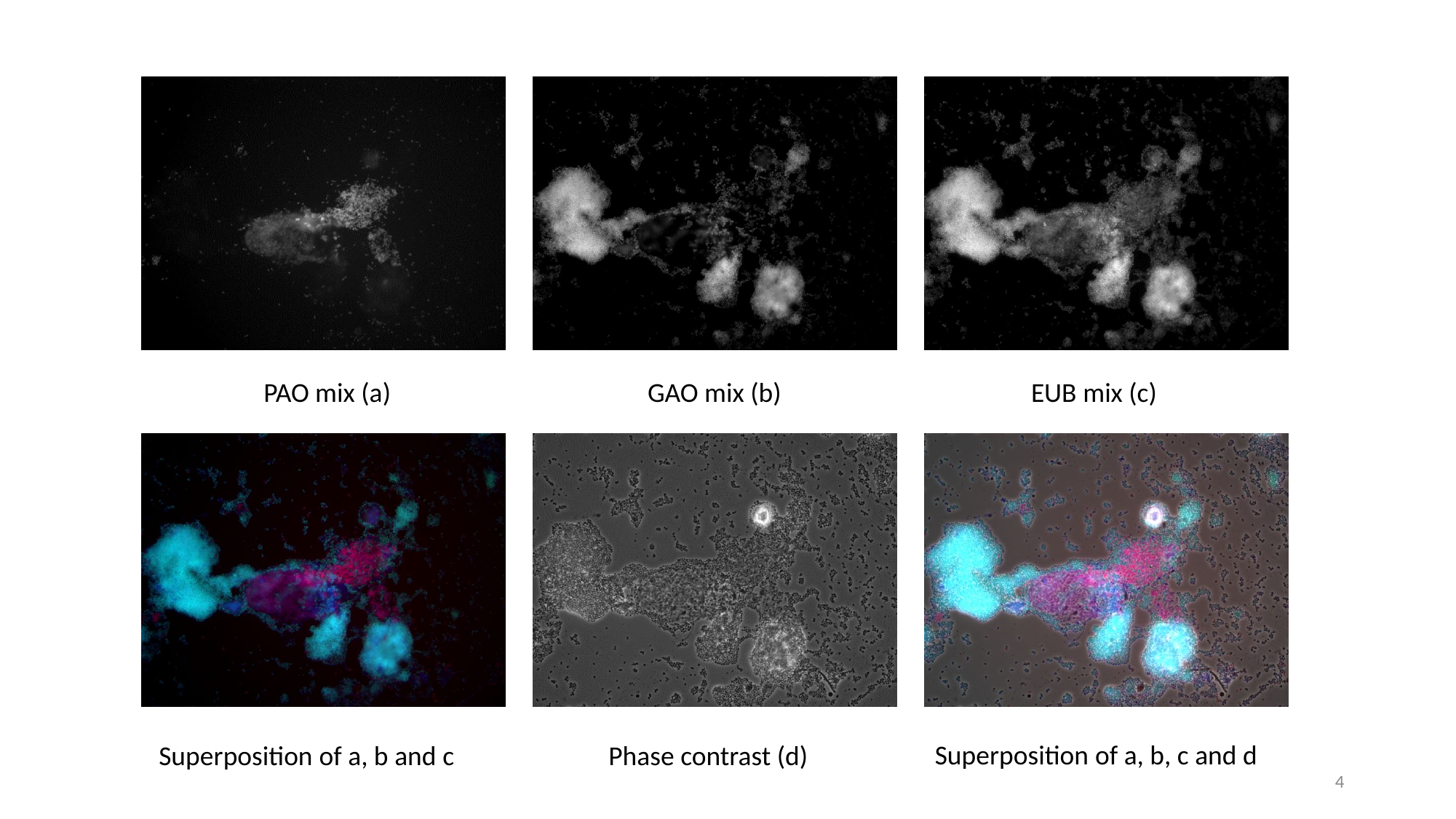

PAO mix (a)
GAO mix (b)
EUB mix (c)
Superposition of a, b, c and d
Superposition of a, b and c
Phase contrast (d)
4

## Slide 5
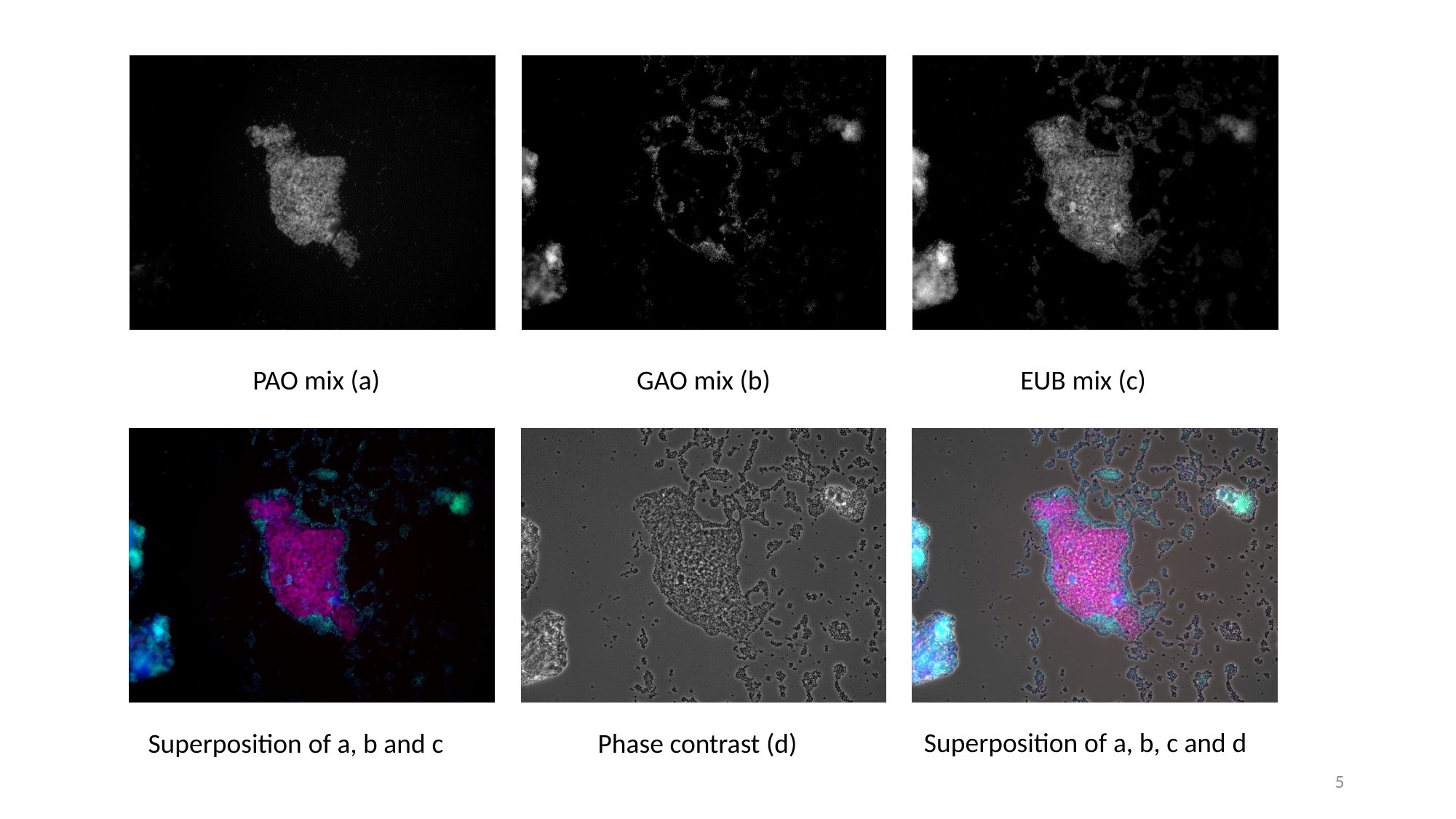

PAO mix (a)
GAO mix (b)
EUB mix (c)
Superposition of a, b, c and d
Superposition of a, b and c
Phase contrast (d)
5

## Slide 6
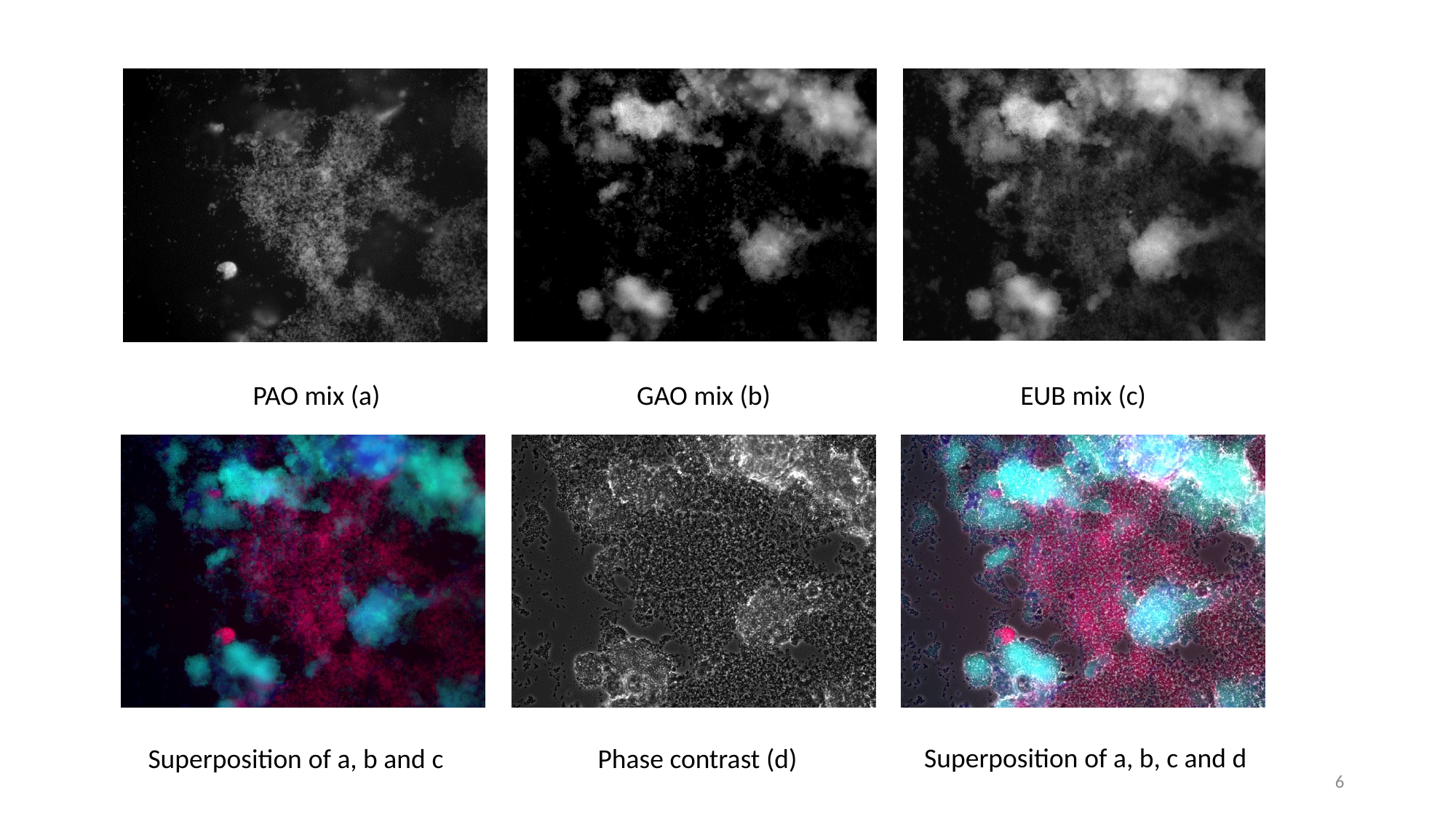

PAO mix (a)
GAO mix (b)
EUB mix (c)
Superposition of a, b, c and d
Superposition of a, b and c
Phase contrast (d)
6

## Slide 7
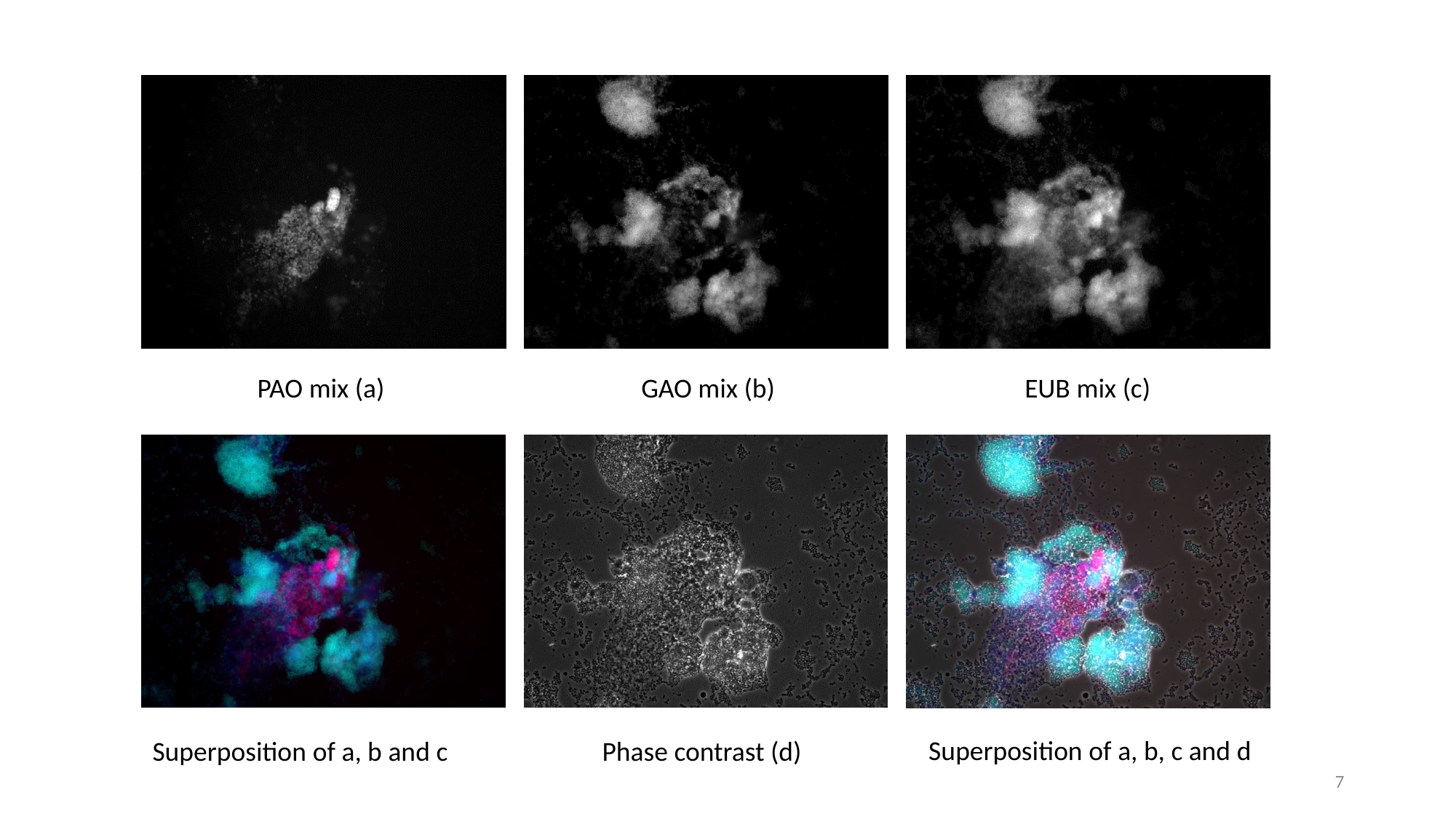

PAO mix (a)
GAO mix (b)
EUB mix (c)
Superposition of a, b, c and d
Superposition of a, b and c
Phase contrast (d)
7

## Slide 8
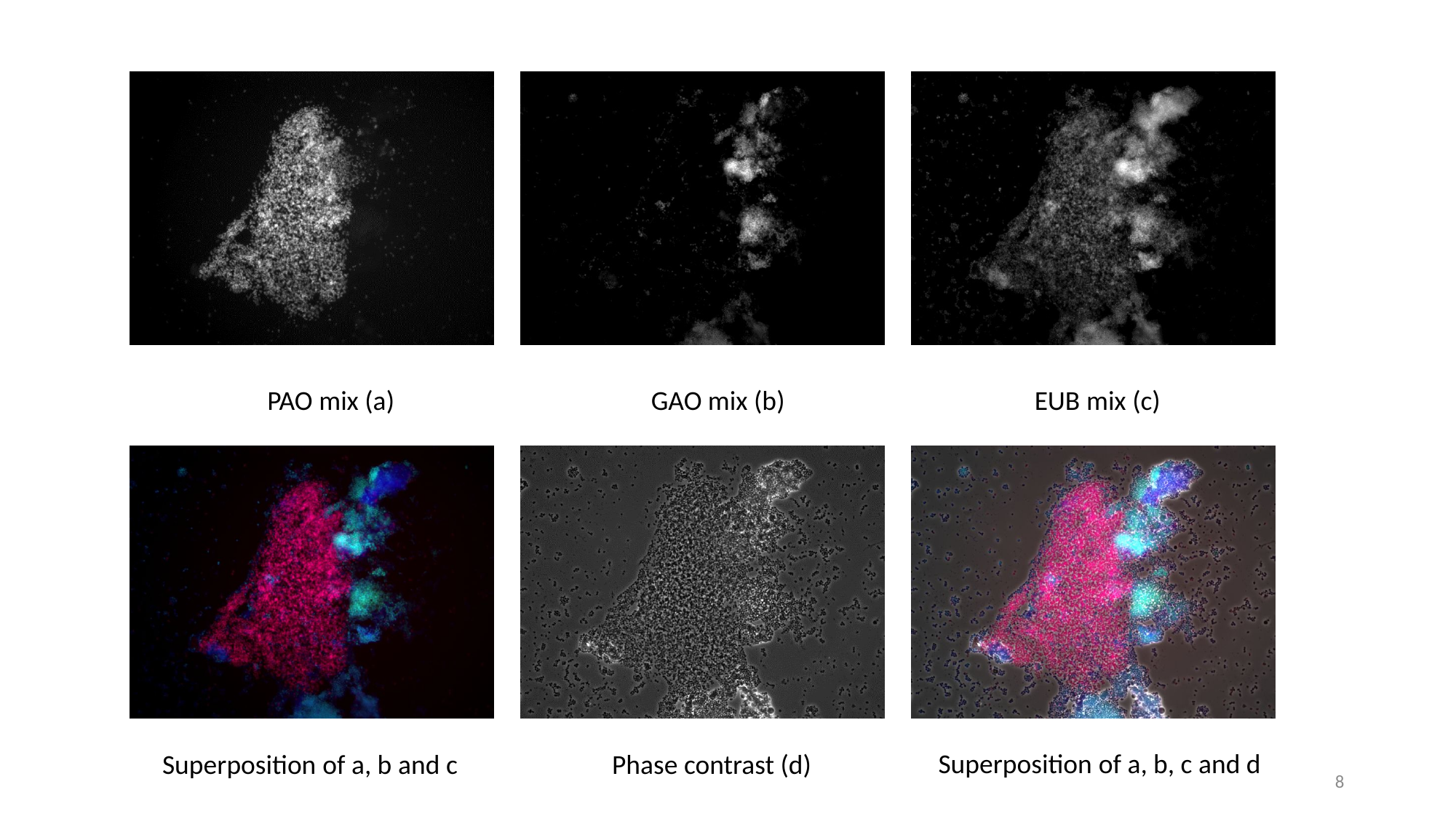

PAO mix (a)
GAO mix (b)
EUB mix (c)
Superposition of a, b, c and d
Superposition of a, b and c
Phase contrast (d)
8

## Slide 9
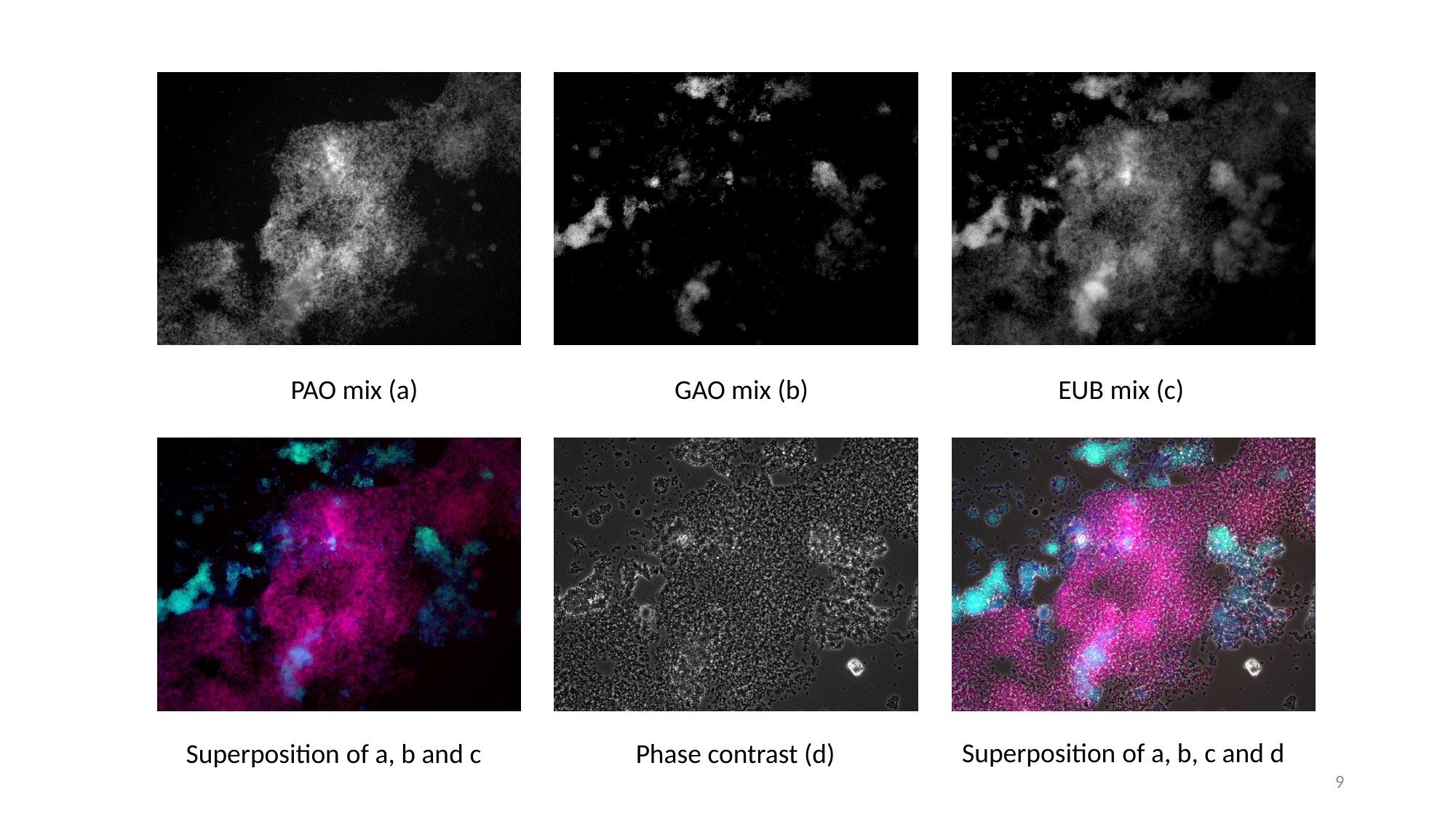

PAO mix (a)
GAO mix (b)
EUB mix (c)
Superposition of a, b, c and d
Superposition of a, b and c
Phase contrast (d)
9

## Slide 10
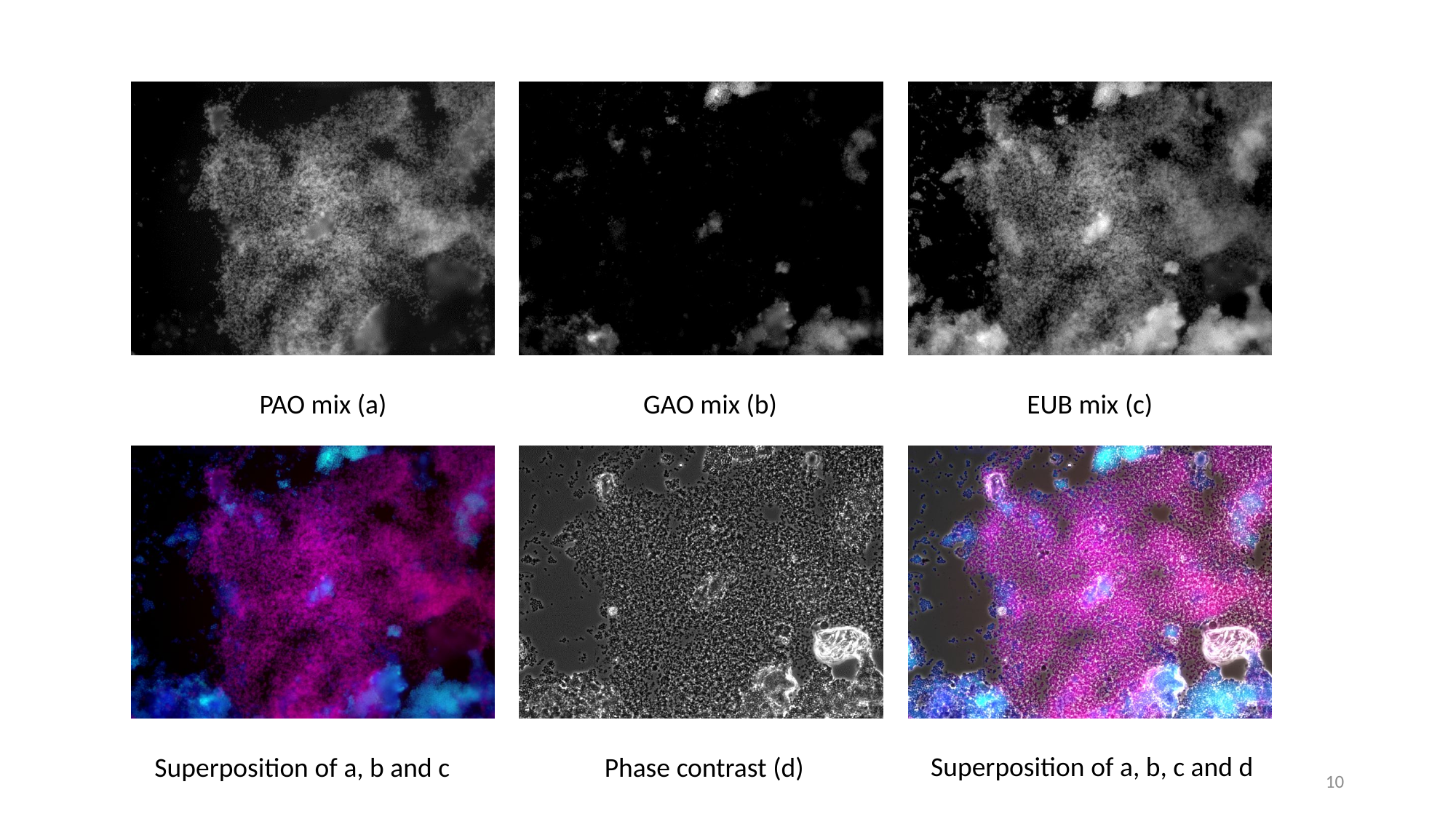

PAO mix (a)
GAO mix (b)
EUB mix (c)
Superposition of a, b, c and d
Superposition of a, b and c
Phase contrast (d)
10

## Slide 11
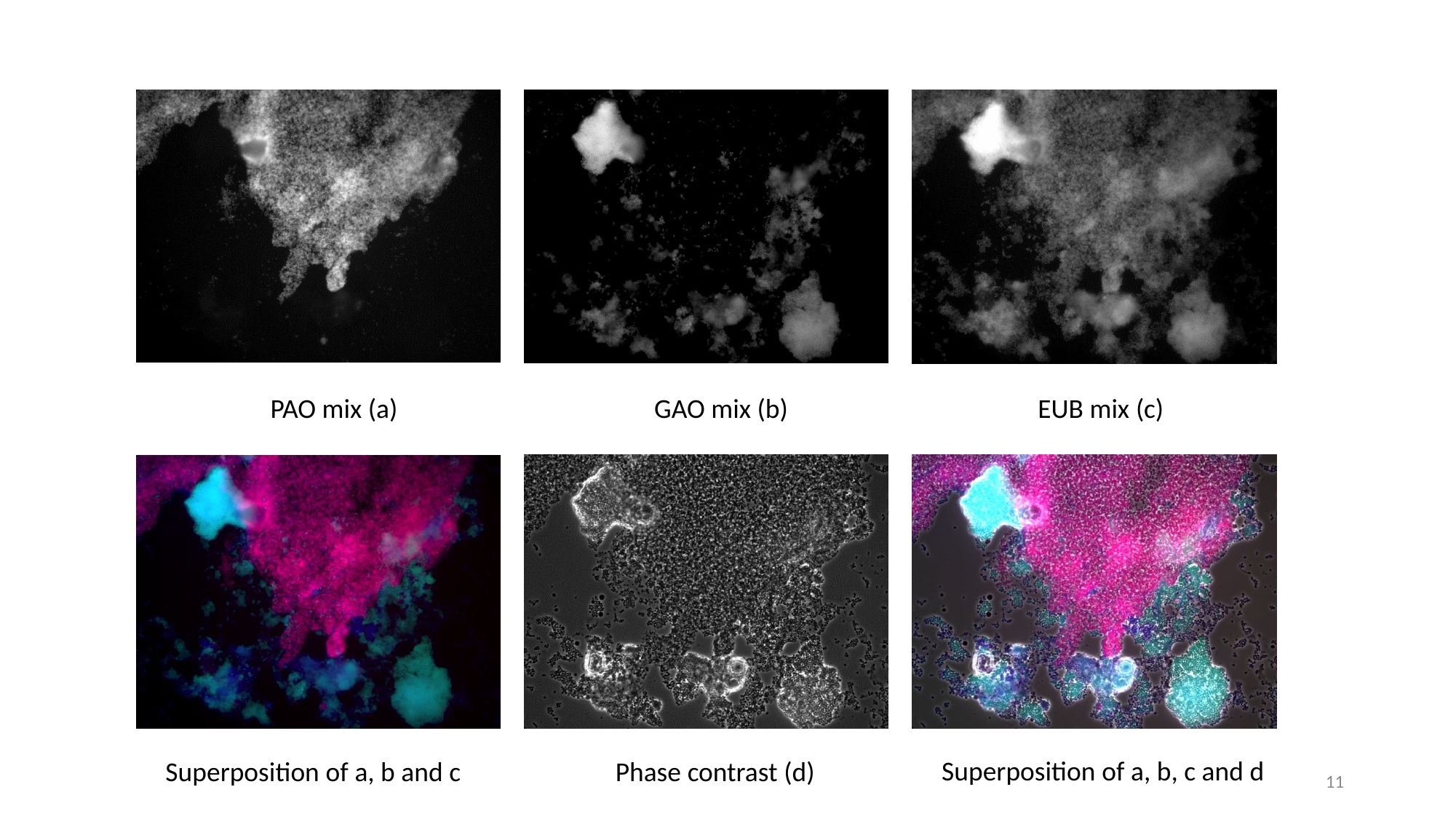

PAO mix (a)
GAO mix (b)
EUB mix (c)
Superposition of a, b, c and d
Superposition of a, b and c
Phase contrast (d)
11

## Slide 12
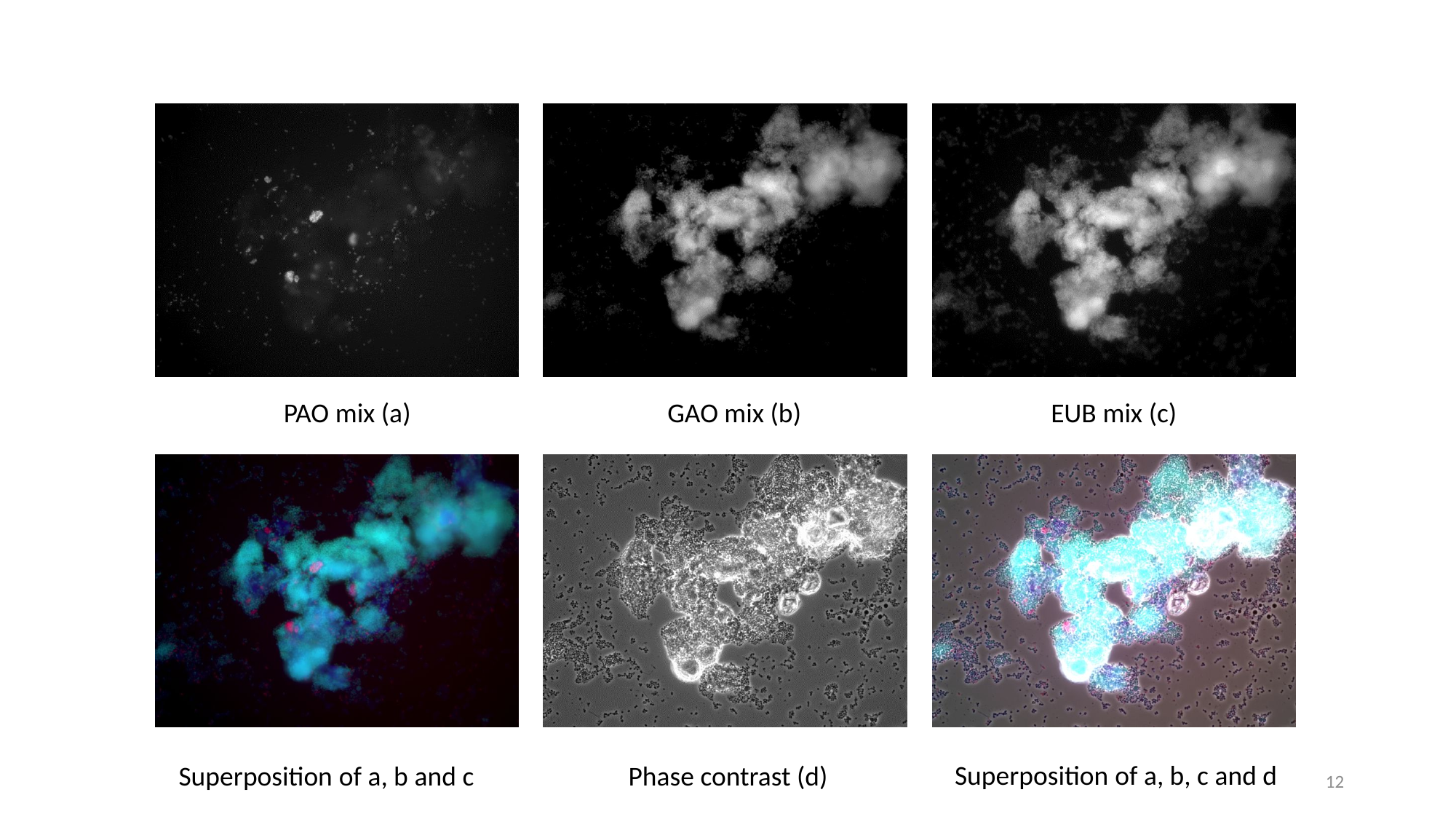

PAO mix (a)
GAO mix (b)
EUB mix (c)
Superposition of a, b, c and d
Superposition of a, b and c
Phase contrast (d)
12

## Slide 13
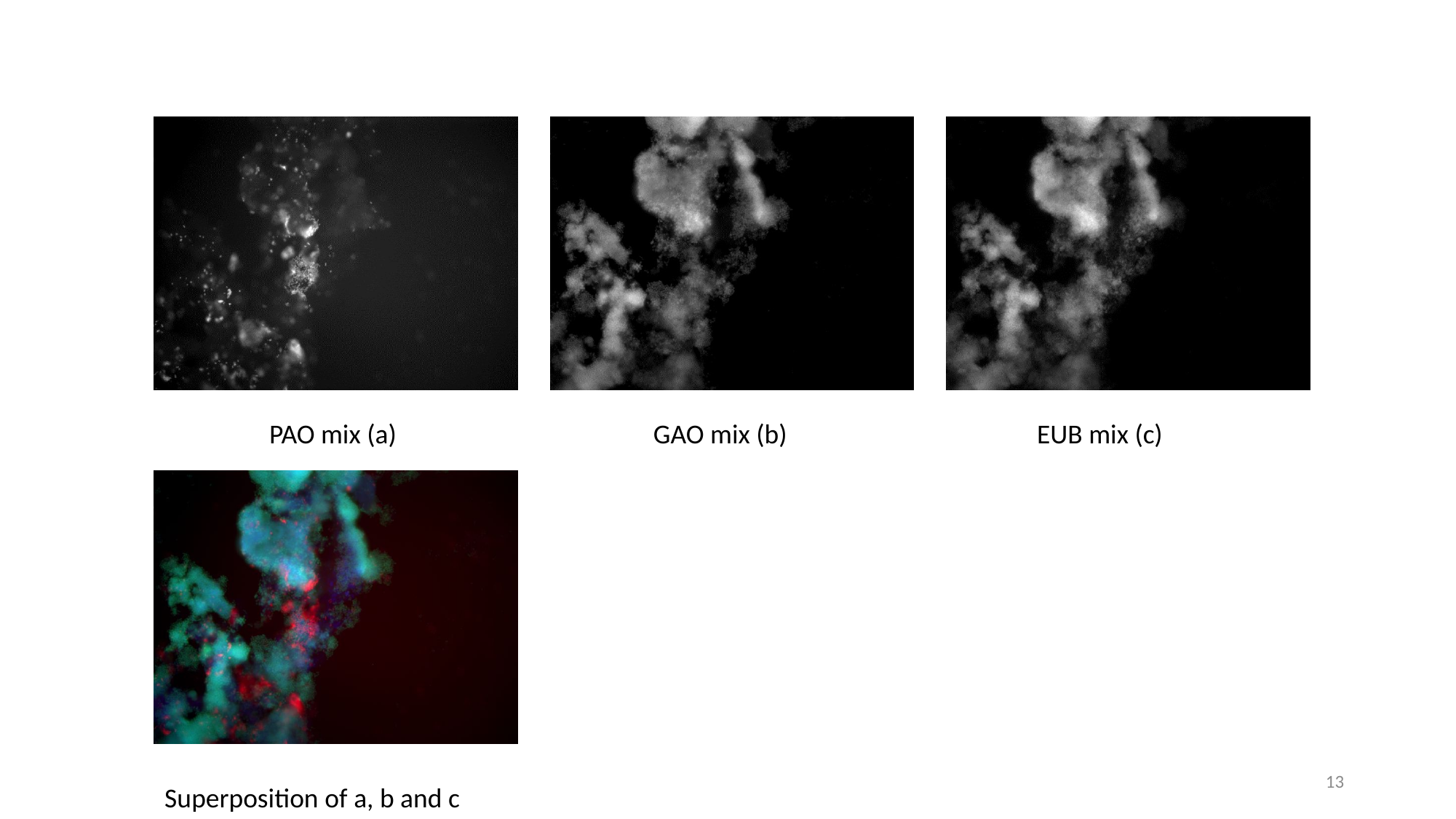

PAO mix (a)
GAO mix (b)
EUB mix (c)
13
Superposition of a, b and c

## Slide 14
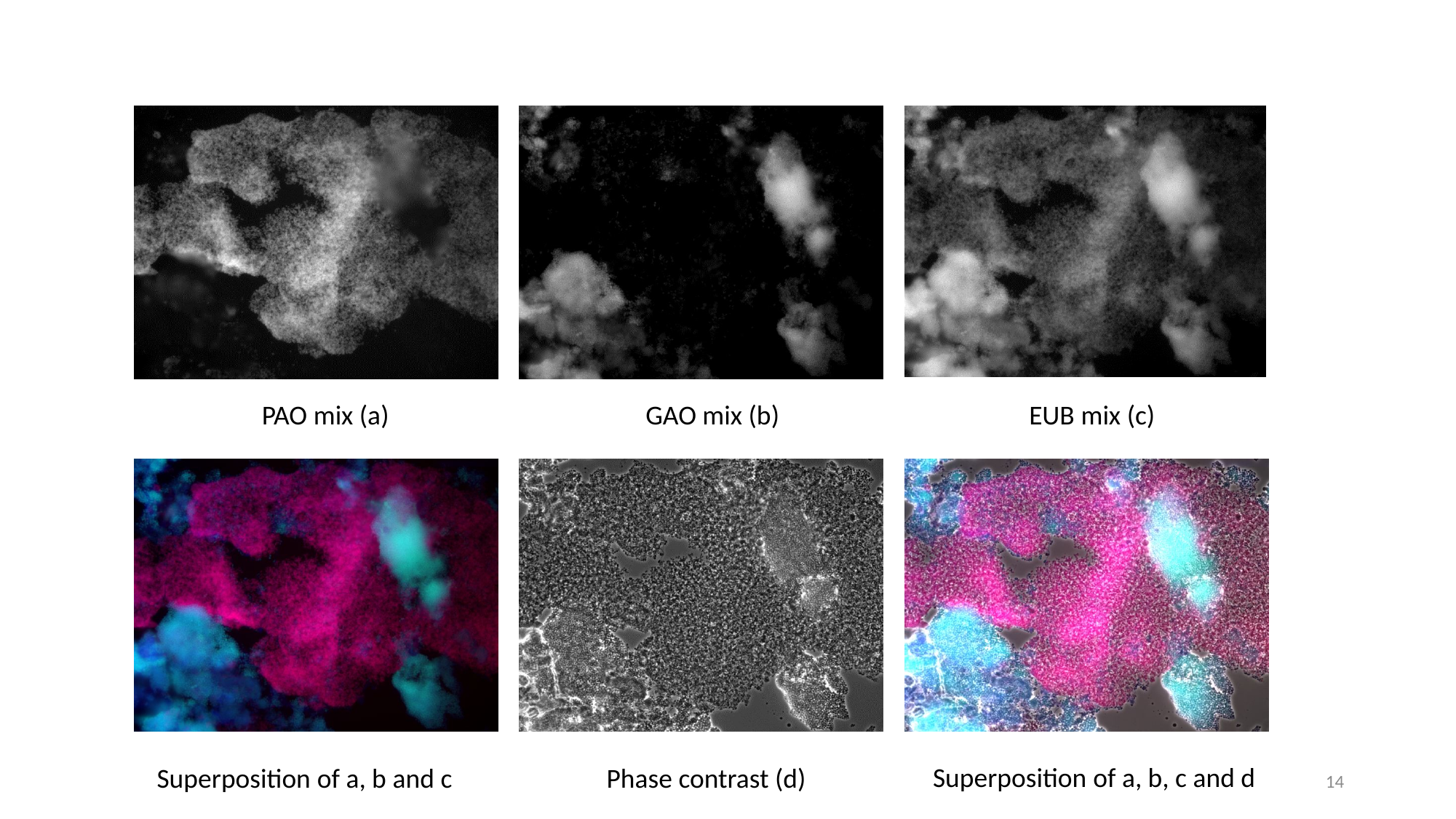

PAO mix (a)
GAO mix (b)
EUB mix (c)
Superposition of a, b, c and d
Superposition of a, b and c
Phase contrast (d)
14

## Slide 15
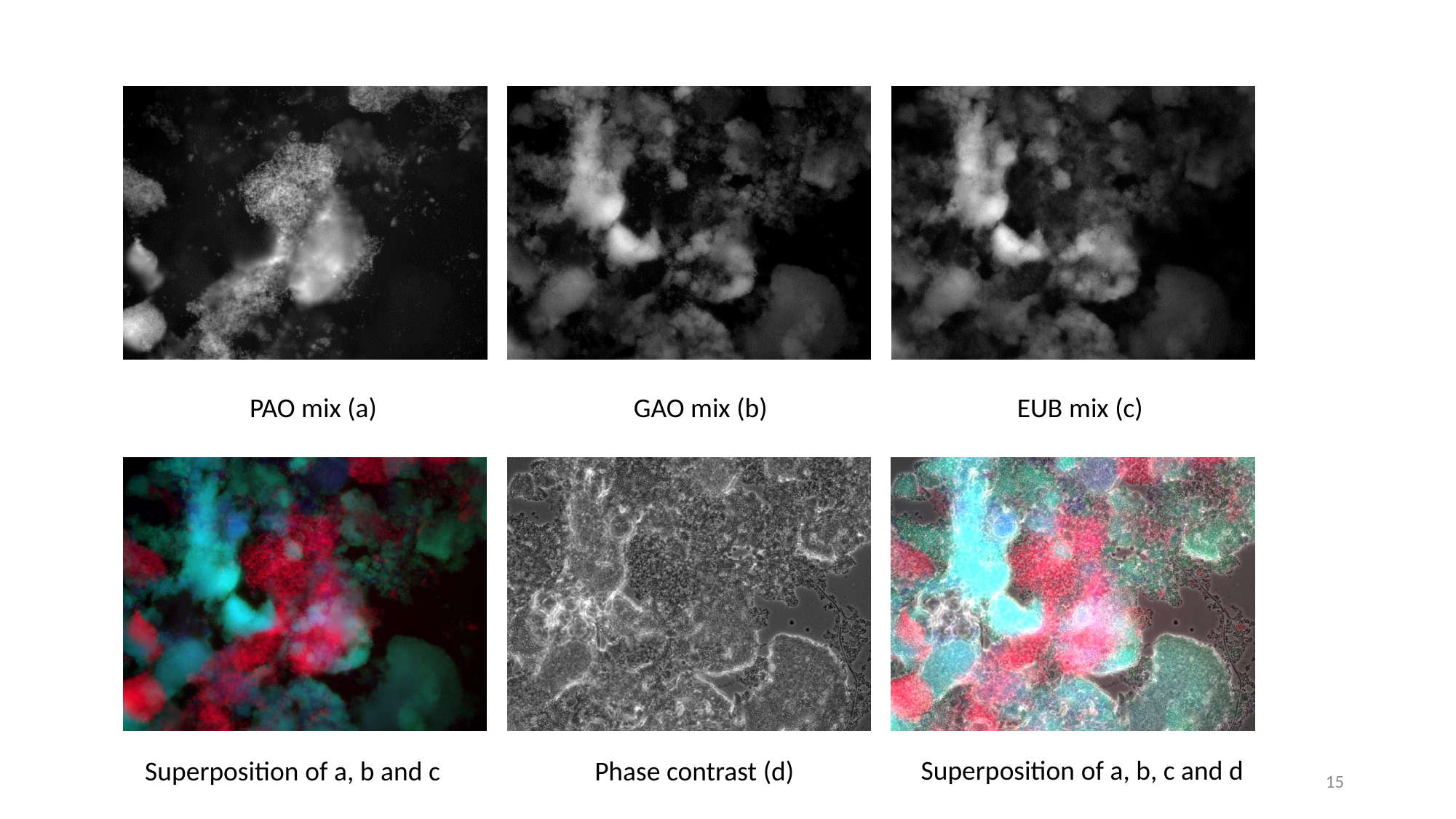

PAO mix (a)
GAO mix (b)
EUB mix (c)
Superposition of a, b, c and d
Superposition of a, b and c
Phase contrast (d)
15

## Slide 16
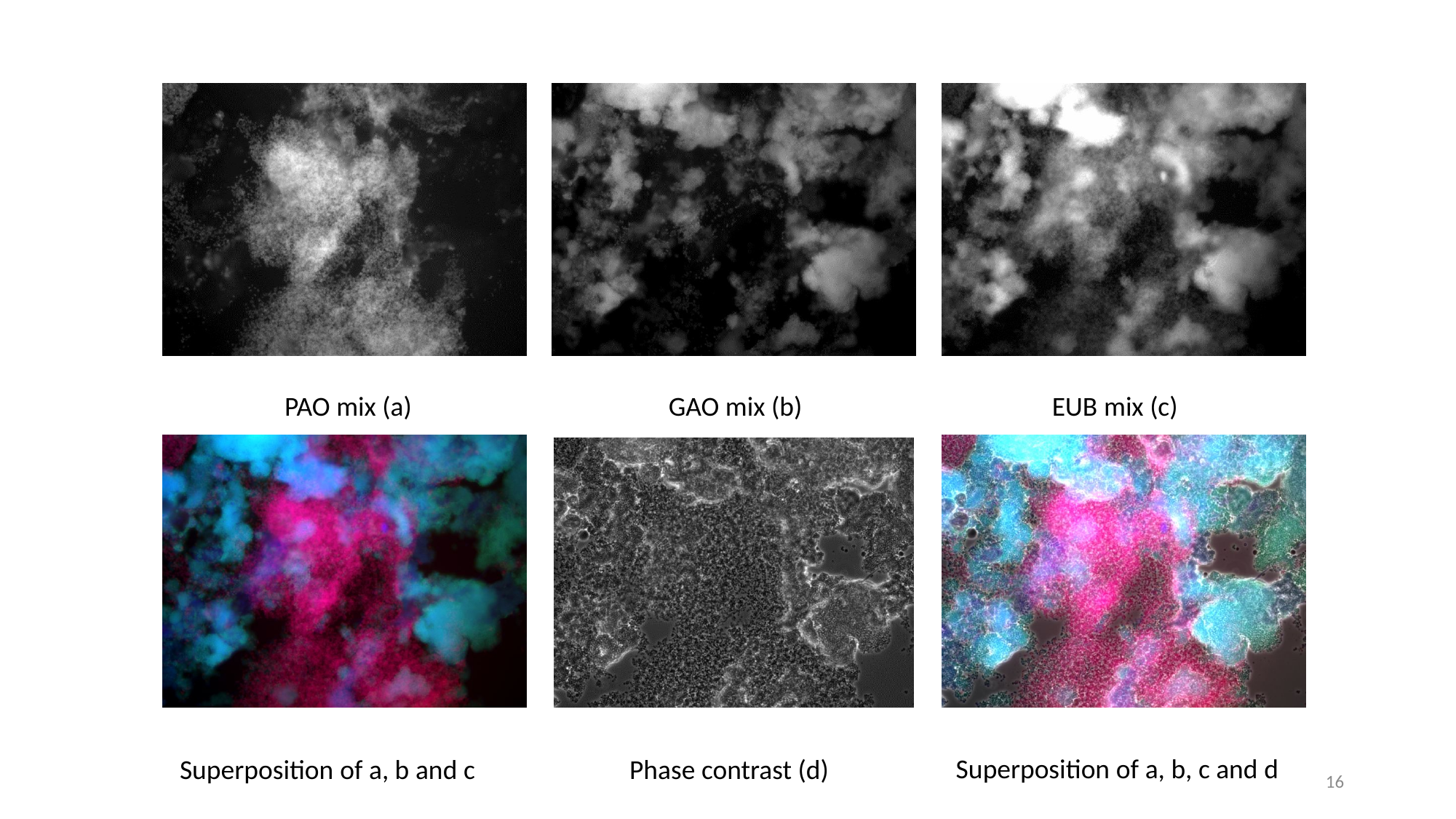

PAO mix (a)
GAO mix (b)
EUB mix (c)
Superposition of a, b, c and d
Superposition of a, b and c
Phase contrast (d)
16

## Slide 17
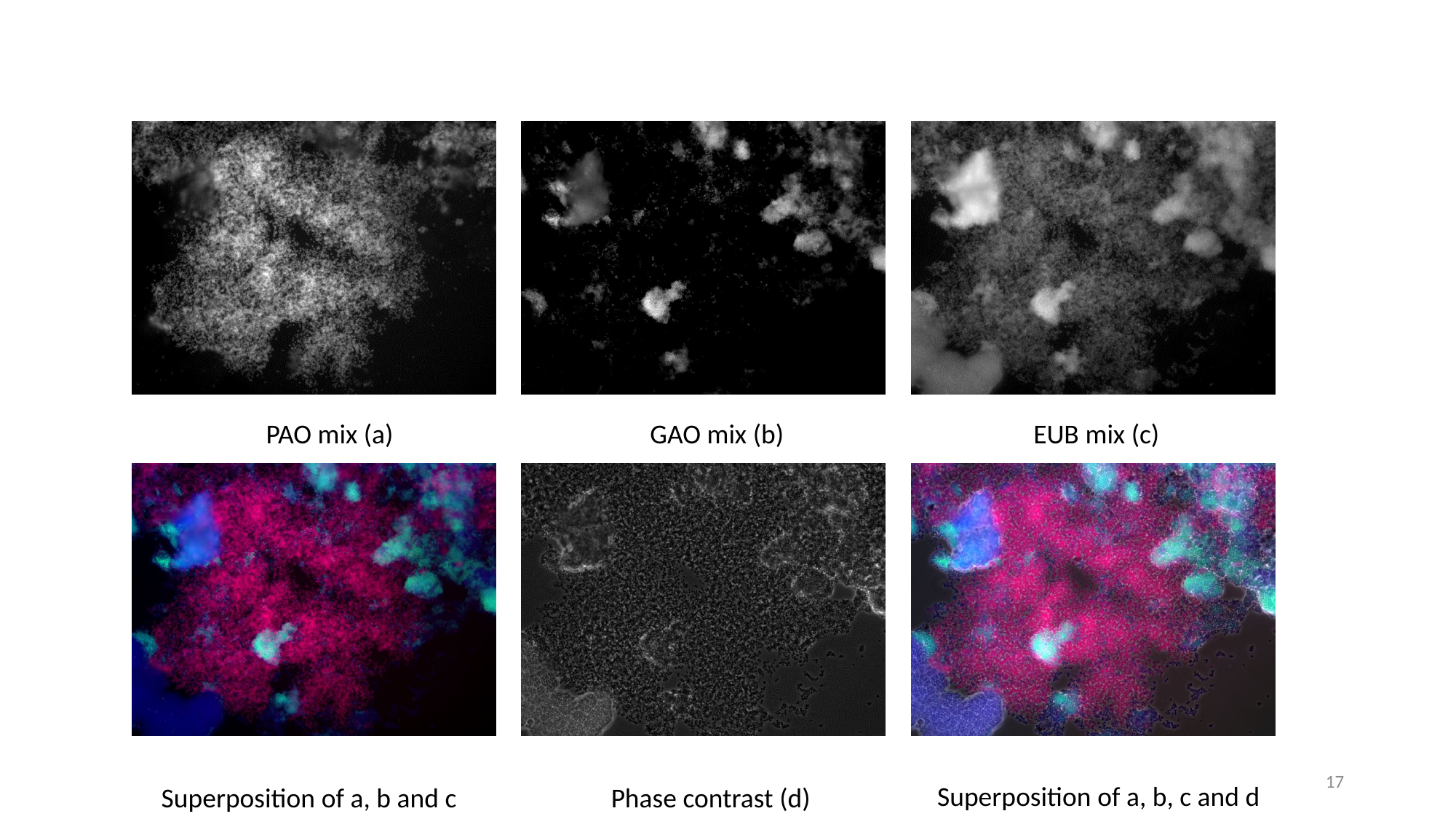

PAO mix (a)
GAO mix (b)
EUB mix (c)
17
Superposition of a, b, c and d
Superposition of a, b and c
Phase contrast (d)

## Slide 18
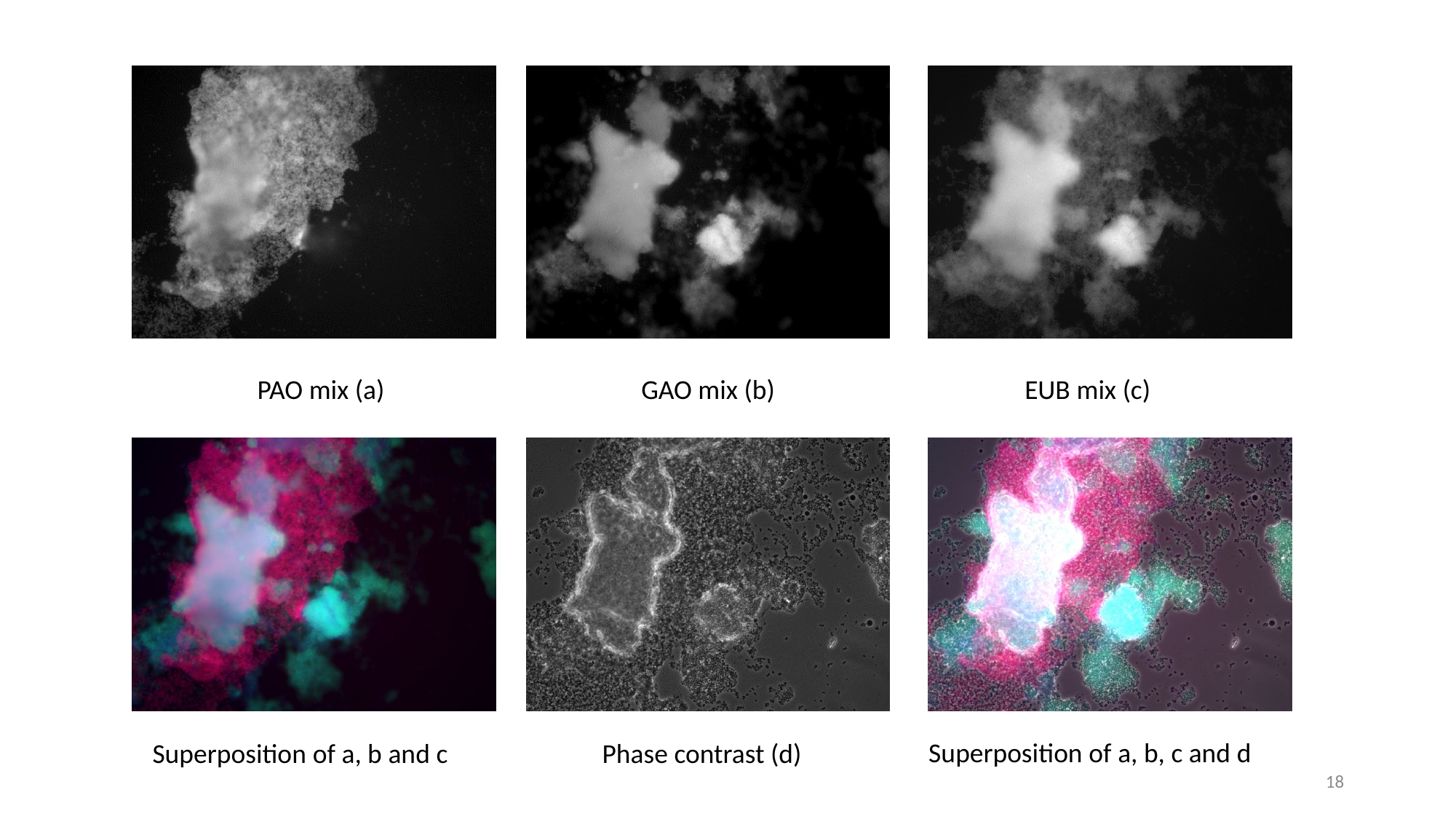

PAO mix (a)
GAO mix (b)
EUB mix (c)
Superposition of a, b, c and d
Superposition of a, b and c
Phase contrast (d)
18

## Slide 19
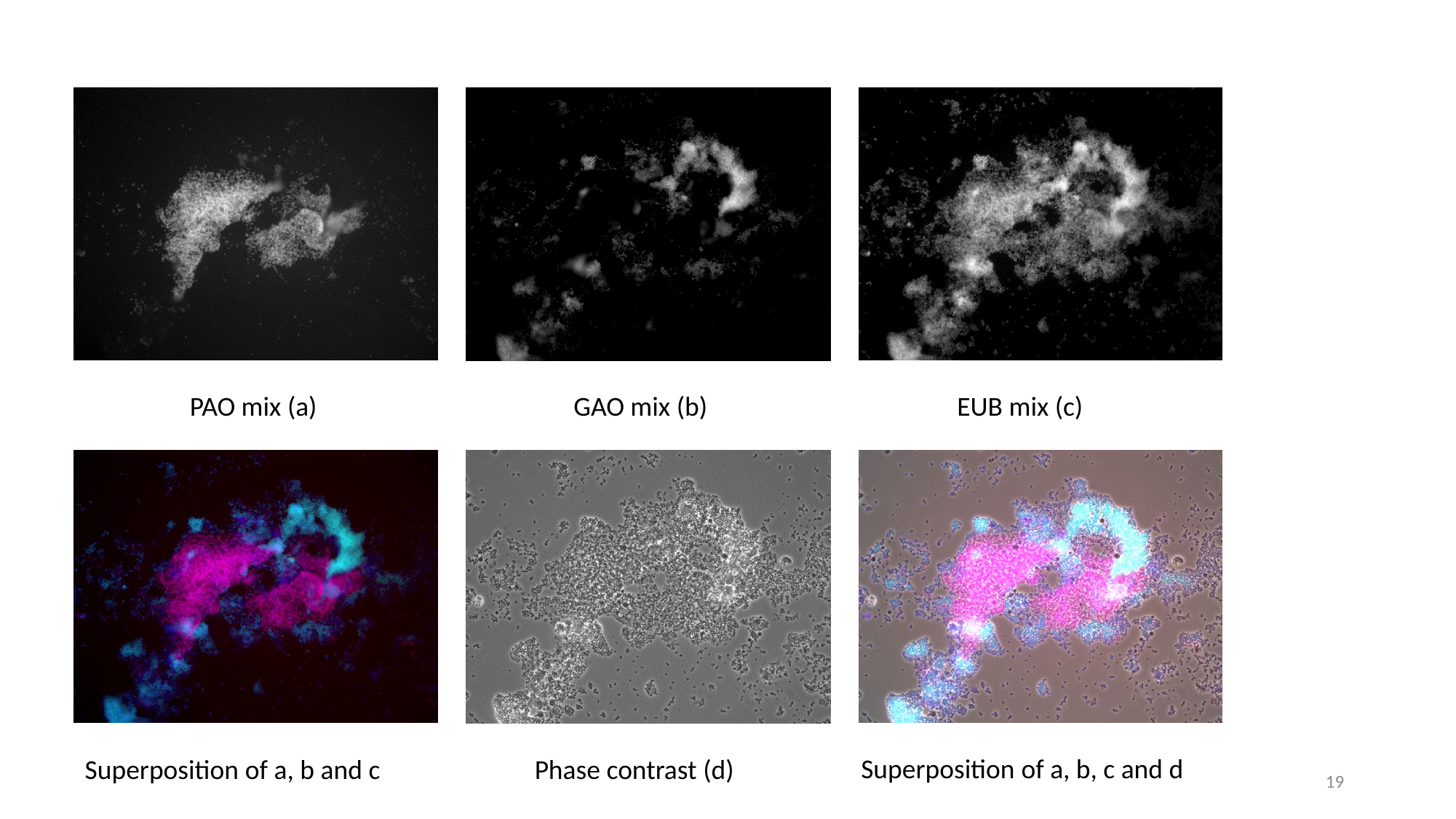

PAO mix (a)
GAO mix (b)
EUB mix (c)
Superposition of a, b, c and d
Superposition of a, b and c
Phase contrast (d)
19

## Slide 20
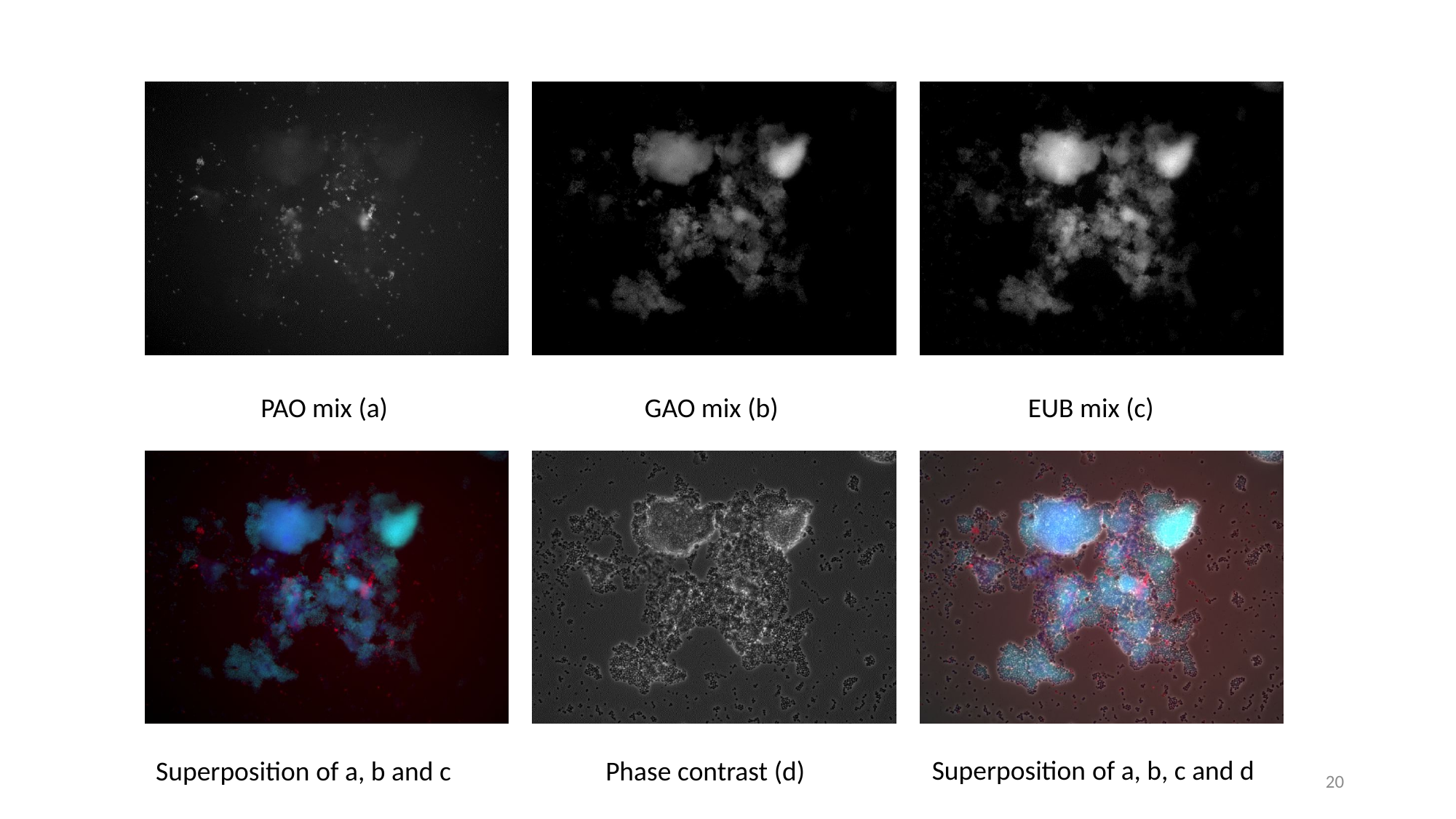

PAO mix (a)
GAO mix (b)
EUB mix (c)
Superposition of a, b, c and d
Superposition of a, b and c
Phase contrast (d)
20

## Slide 21
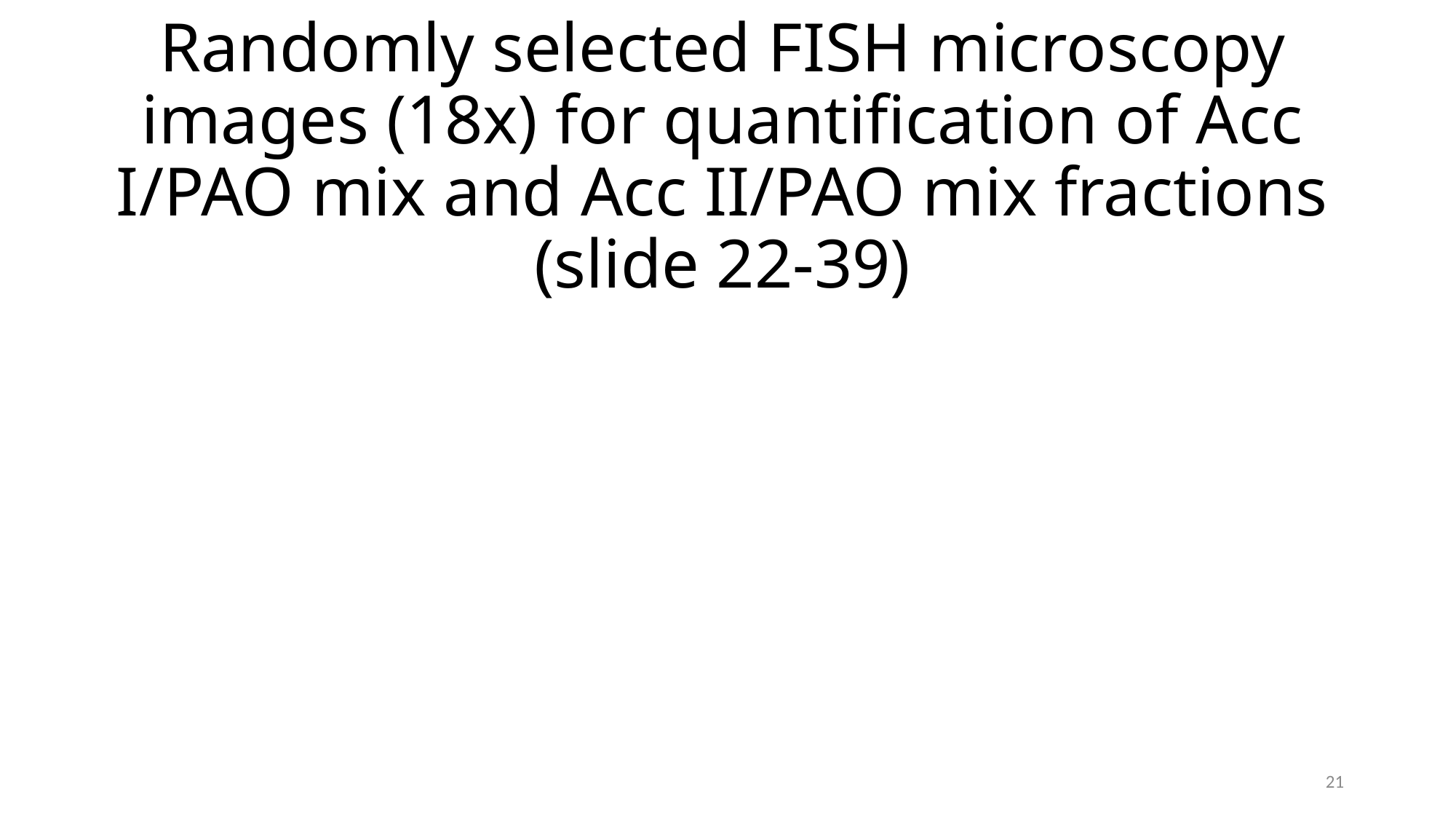

# Randomly selected FISH microscopy images (18x) for quantification of Acc I/PAO mix and Acc II/PAO mix fractions (slide 22-39)
21

## Slide 22
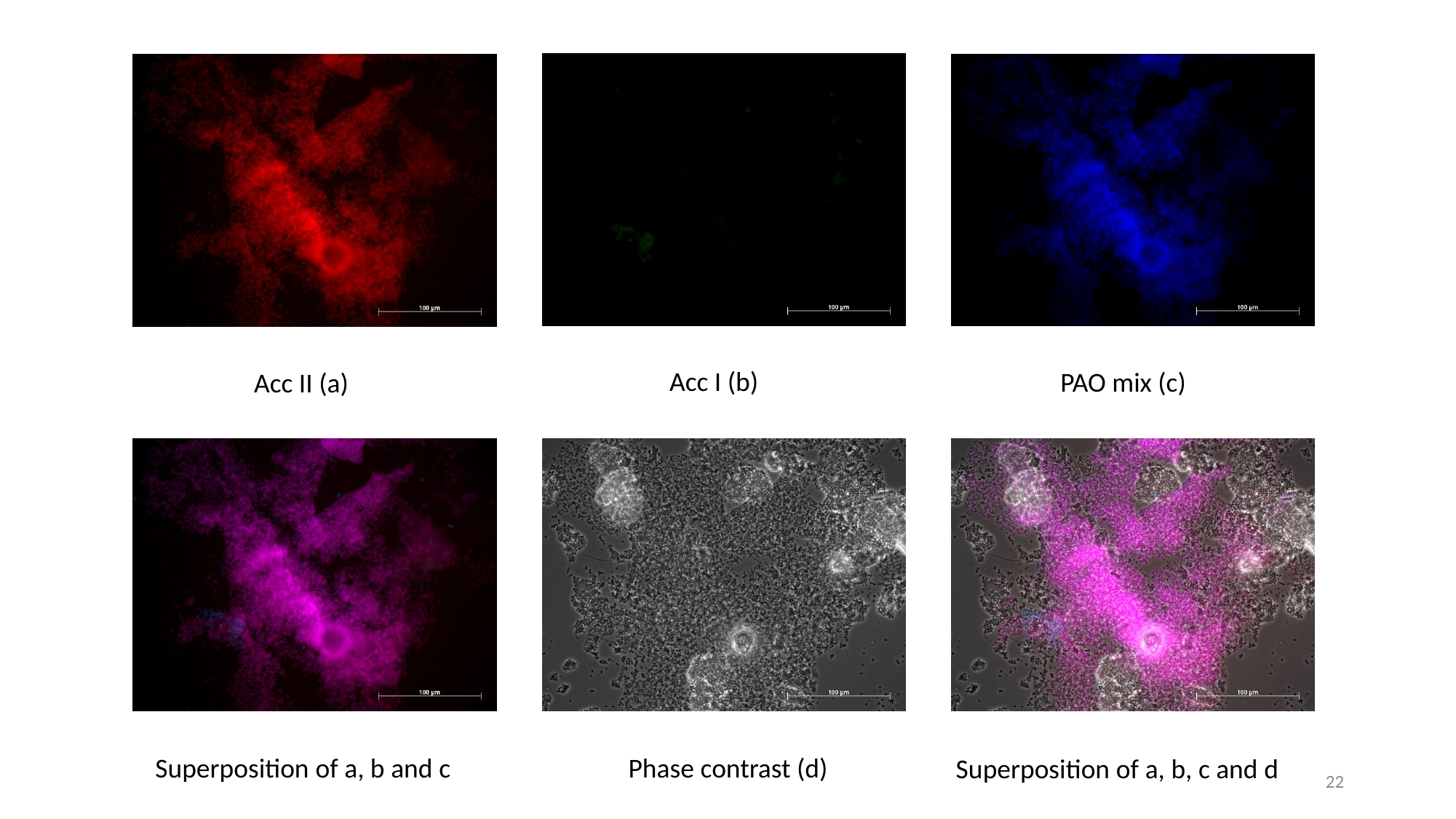

Acc I (b)
PAO mix (c)
Acc II (a)
Superposition of a, b and c
Phase contrast (d)
Superposition of a, b, c and d
22

## Slide 23
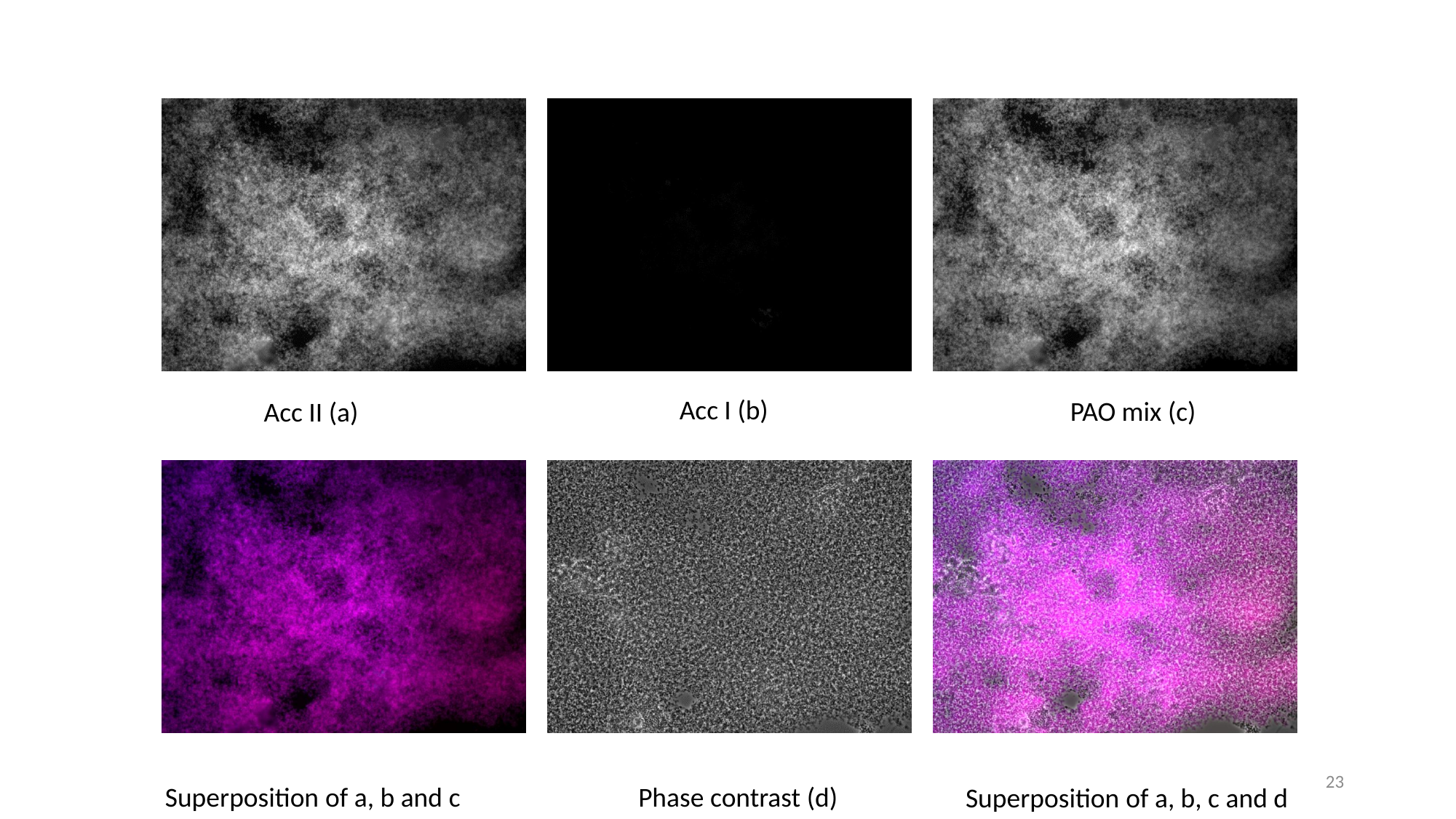

Acc I (b)
PAO mix (c)
Acc II (a)
23
Superposition of a, b and c
Phase contrast (d)
Superposition of a, b, c and d

## Slide 24
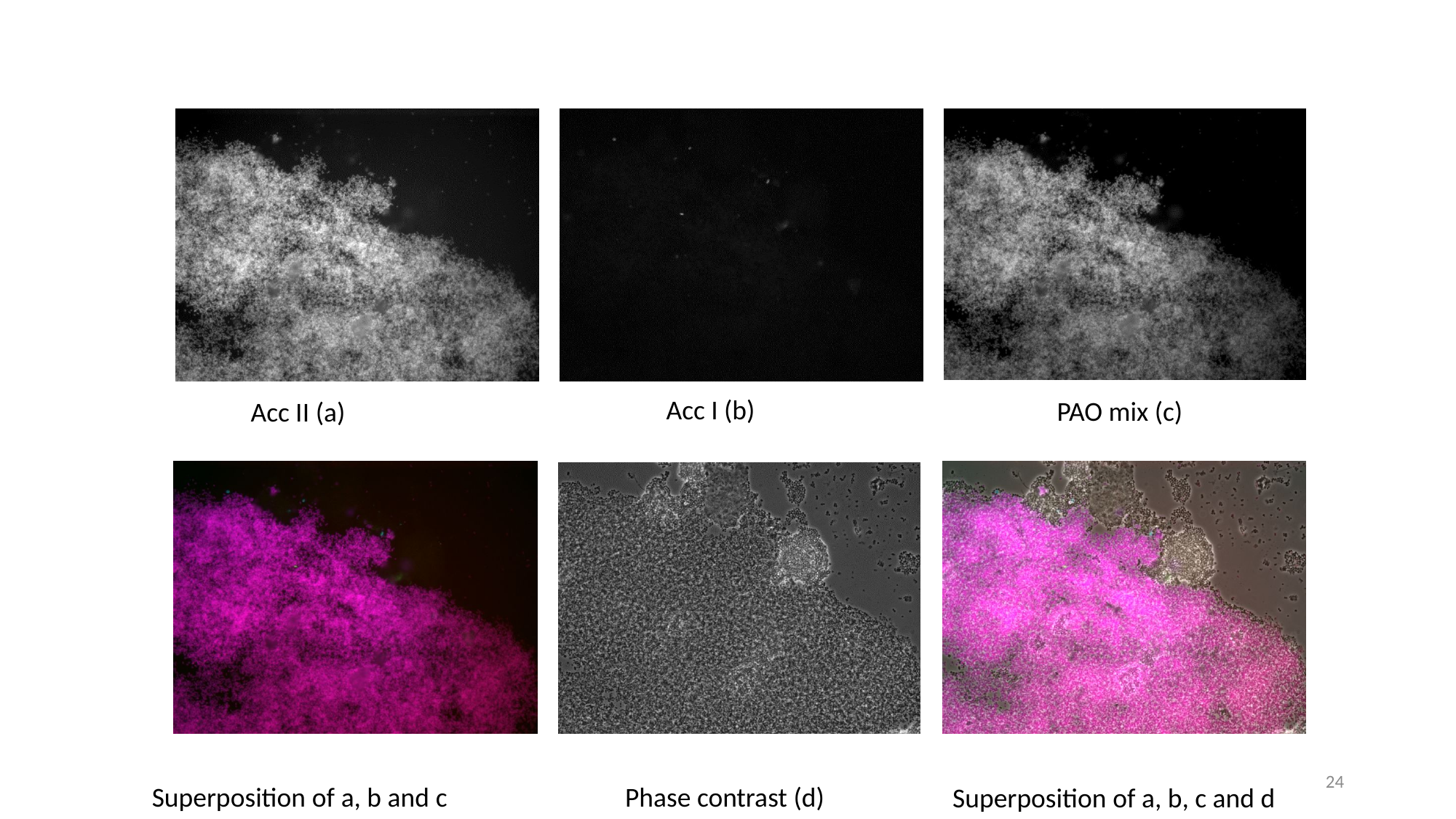

Acc I (b)
PAO mix (c)
Acc II (a)
24
Superposition of a, b and c
Phase contrast (d)
Superposition of a, b, c and d

## Slide 25
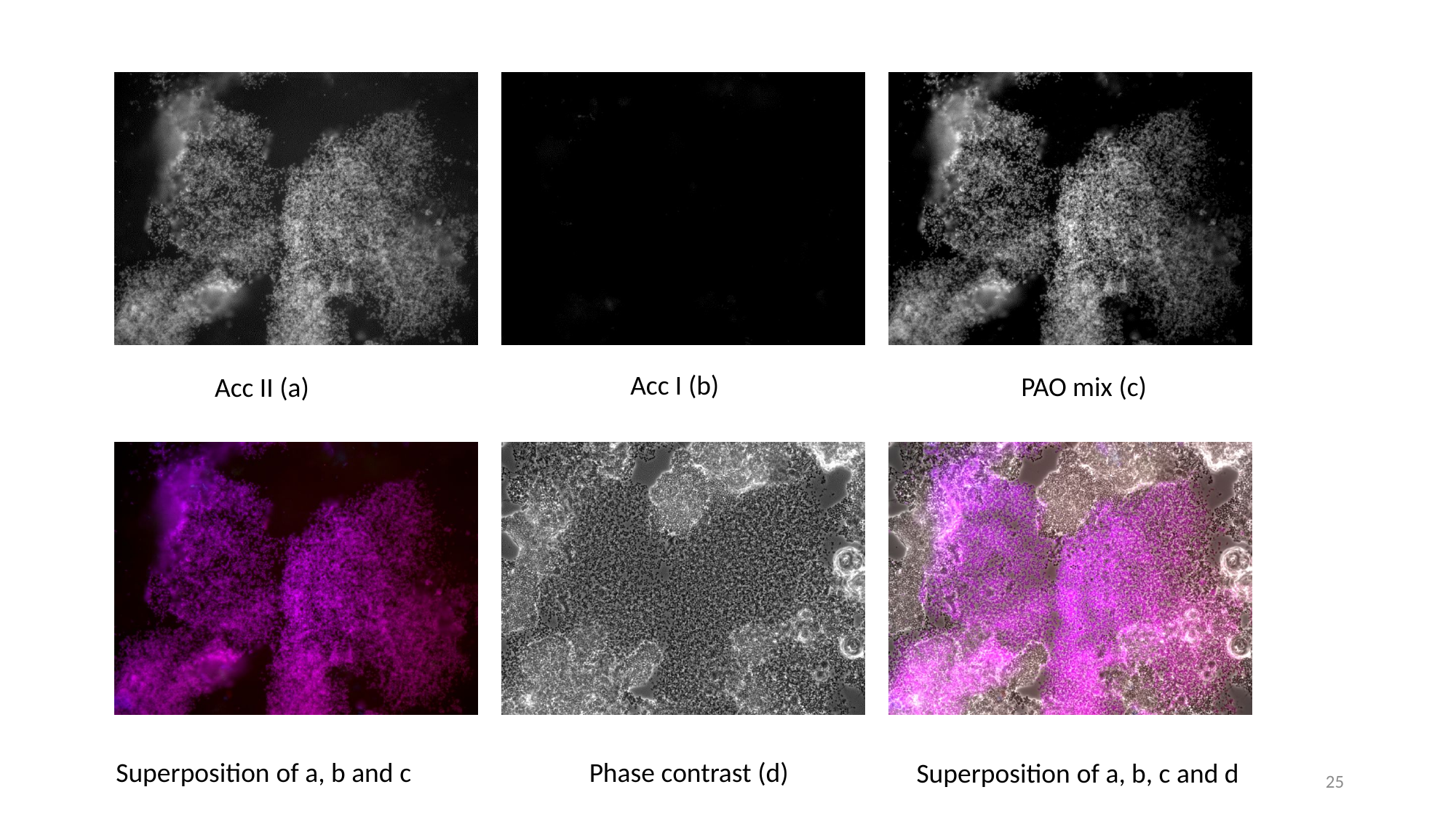

Acc I (b)
PAO mix (c)
Acc II (a)
Superposition of a, b and c
Phase contrast (d)
Superposition of a, b, c and d
25

## Slide 26
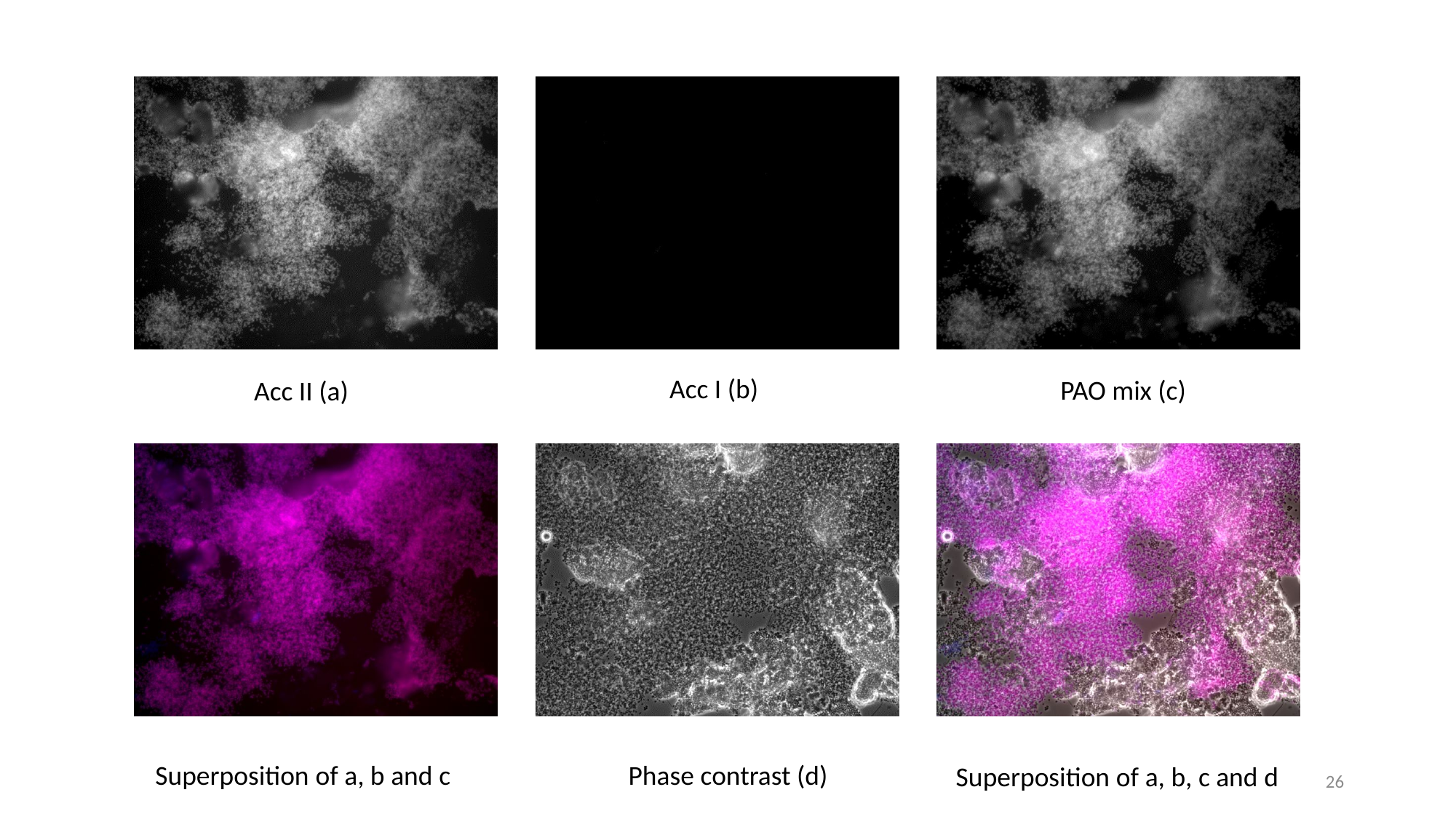

Acc I (b)
PAO mix (c)
Acc II (a)
Superposition of a, b and c
Phase contrast (d)
Superposition of a, b, c and d
26

## Slide 27
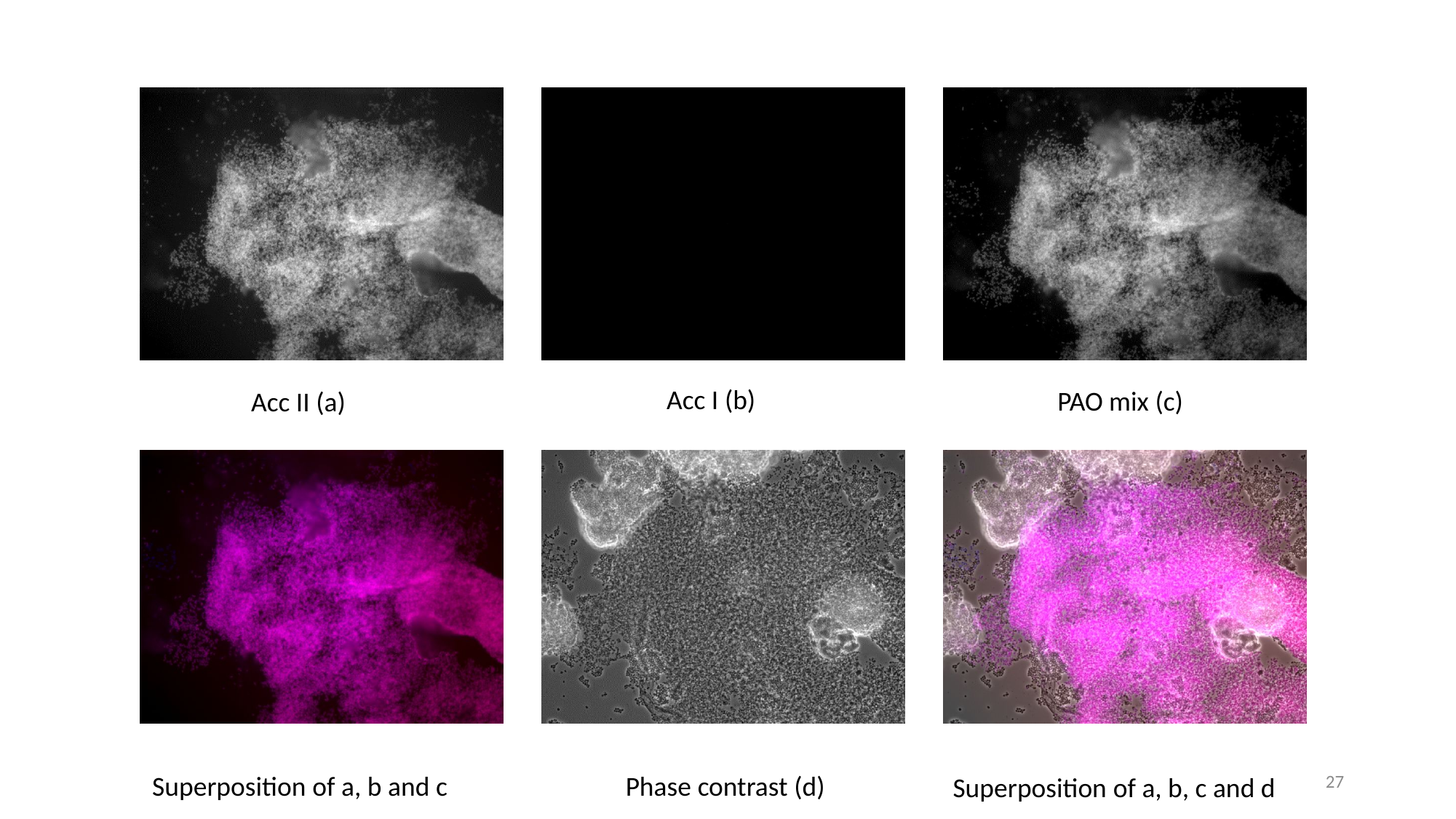

Acc I (b)
PAO mix (c)
Acc II (a)
27
Superposition of a, b and c
Phase contrast (d)
Superposition of a, b, c and d

## Slide 28
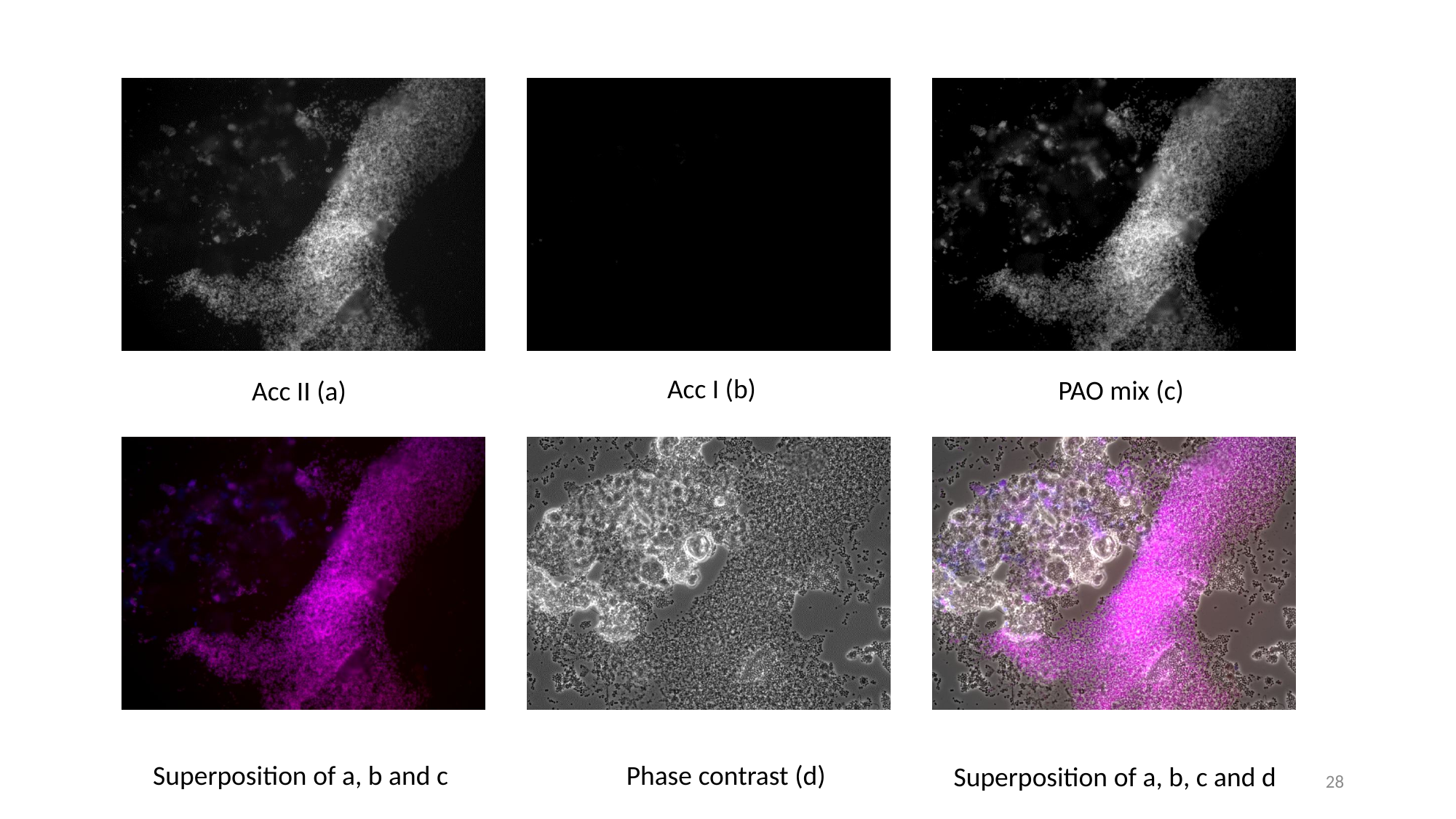

Acc I (b)
PAO mix (c)
Acc II (a)
Superposition of a, b and c
Phase contrast (d)
Superposition of a, b, c and d
28

## Slide 29
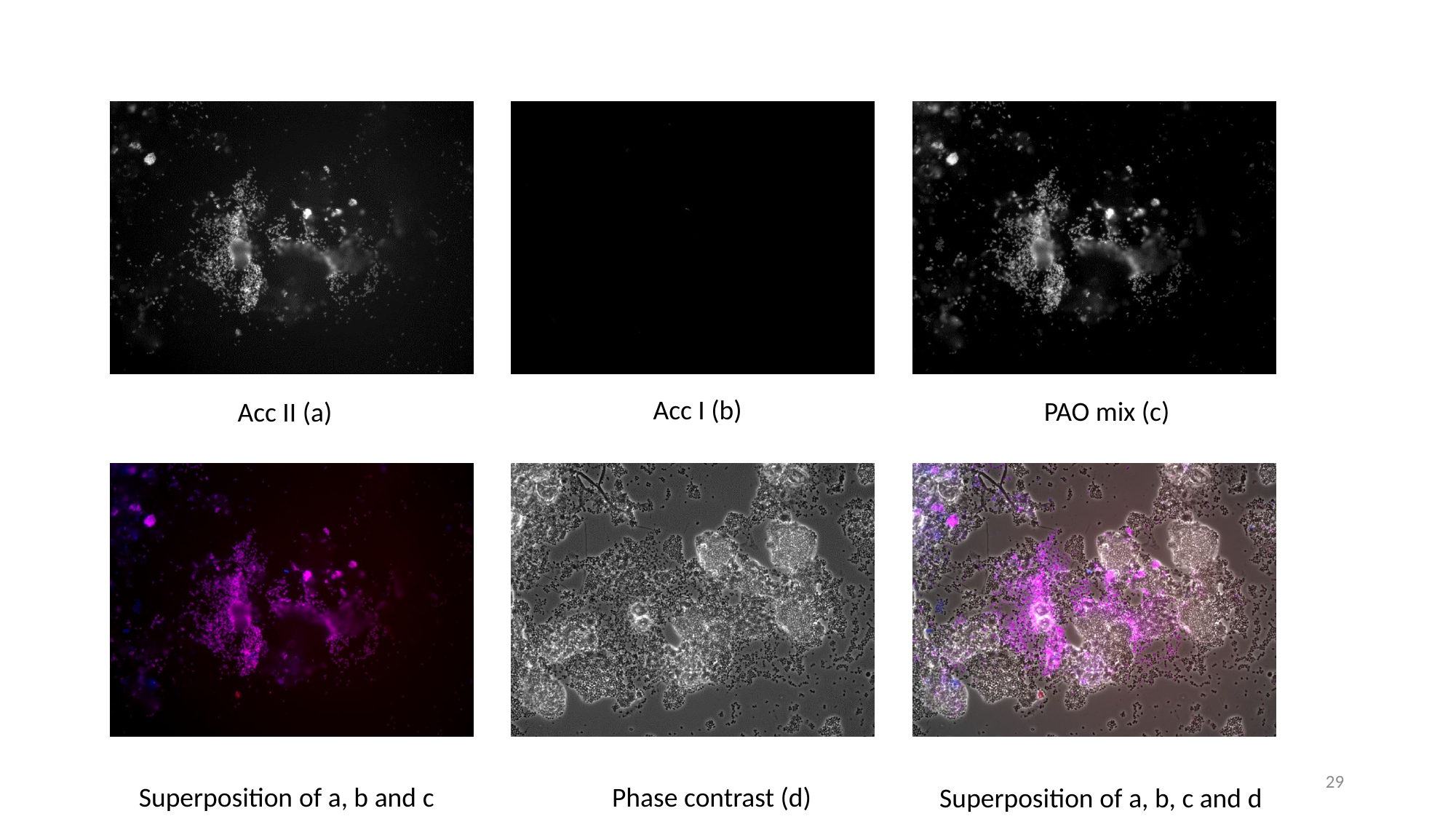

Acc I (b)
PAO mix (c)
Acc II (a)
29
Superposition of a, b and c
Phase contrast (d)
Superposition of a, b, c and d

## Slide 30
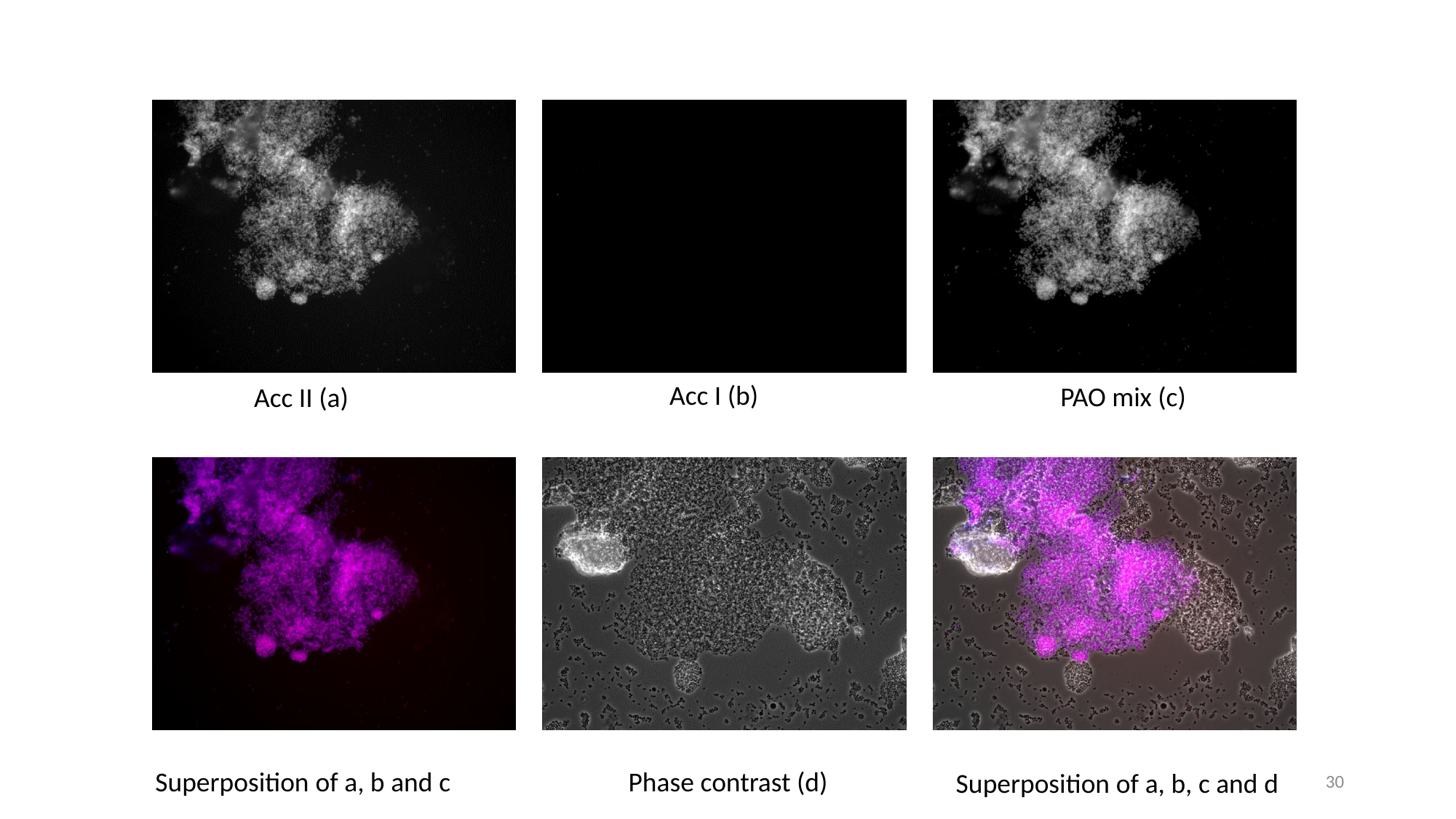

Acc I (b)
PAO mix (c)
Acc II (a)
30
Superposition of a, b and c
Phase contrast (d)
Superposition of a, b, c and d

## Slide 31
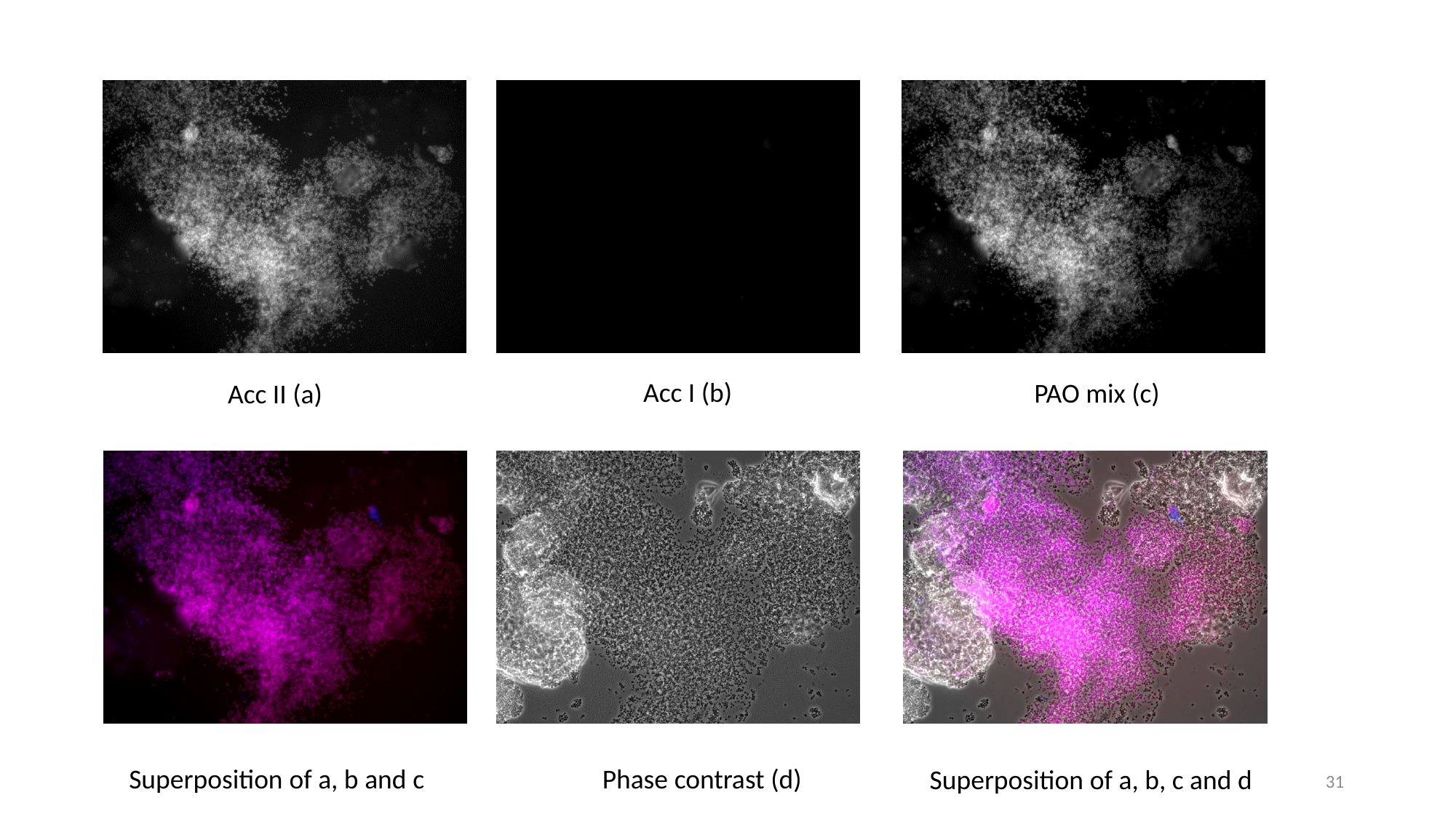

Acc I (b)
PAO mix (c)
Acc II (a)
Superposition of a, b and c
Phase contrast (d)
Superposition of a, b, c and d
31

## Slide 32
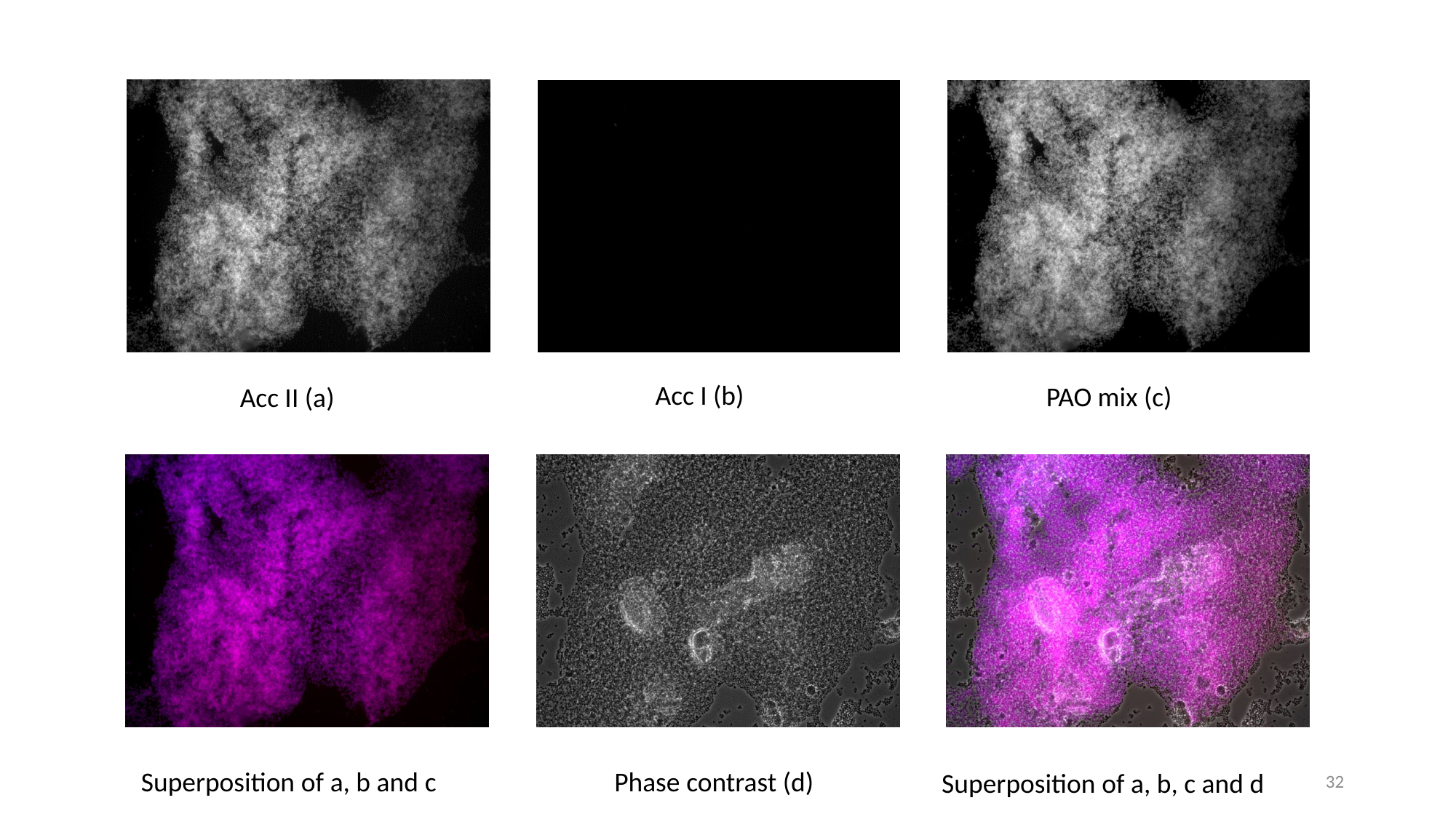

Acc I (b)
PAO mix (c)
Acc II (a)
32
Superposition of a, b and c
Phase contrast (d)
Superposition of a, b, c and d

## Slide 33
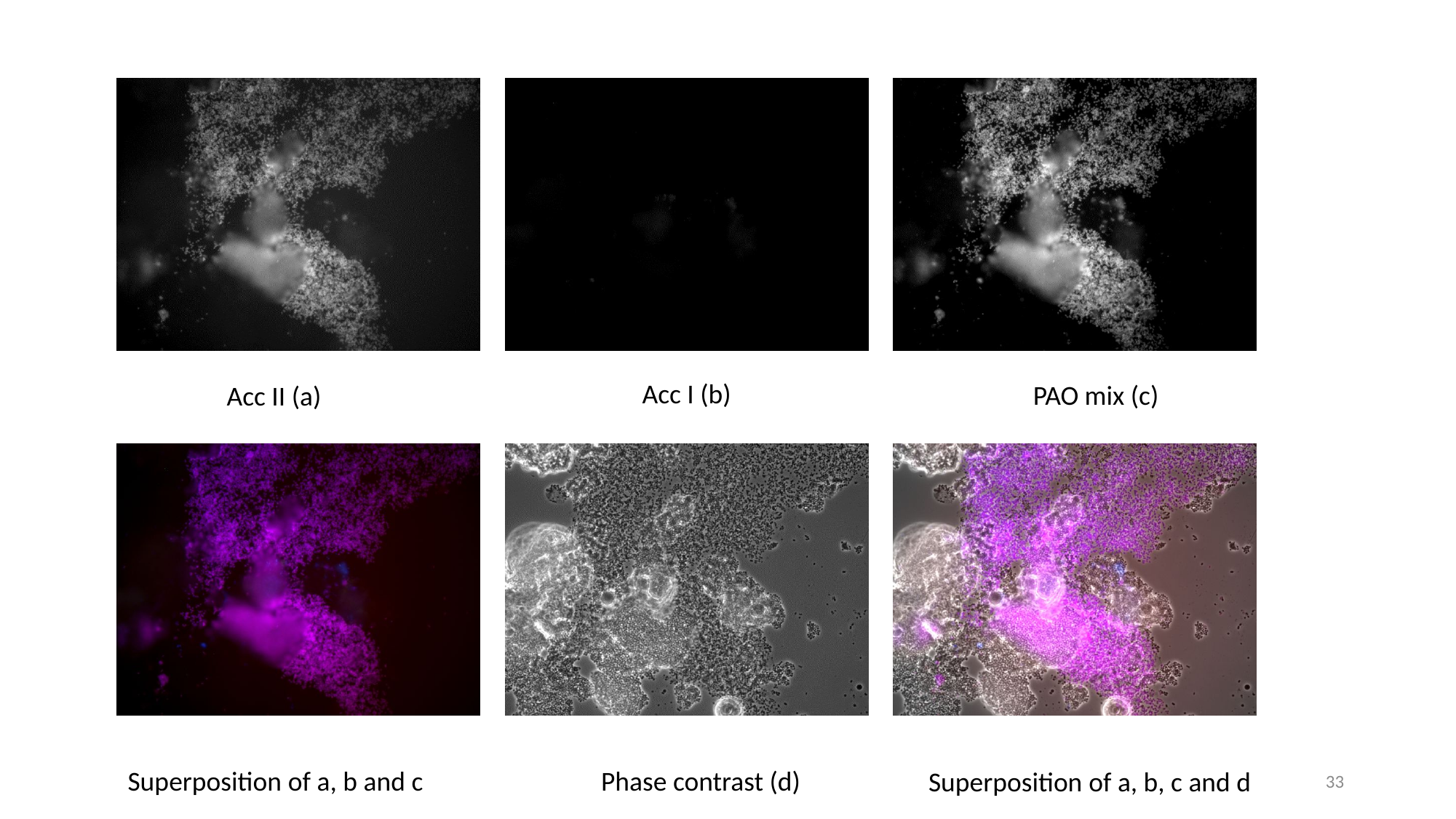

Acc I (b)
PAO mix (c)
Acc II (a)
Superposition of a, b and c
Phase contrast (d)
33
Superposition of a, b, c and d

## Slide 34
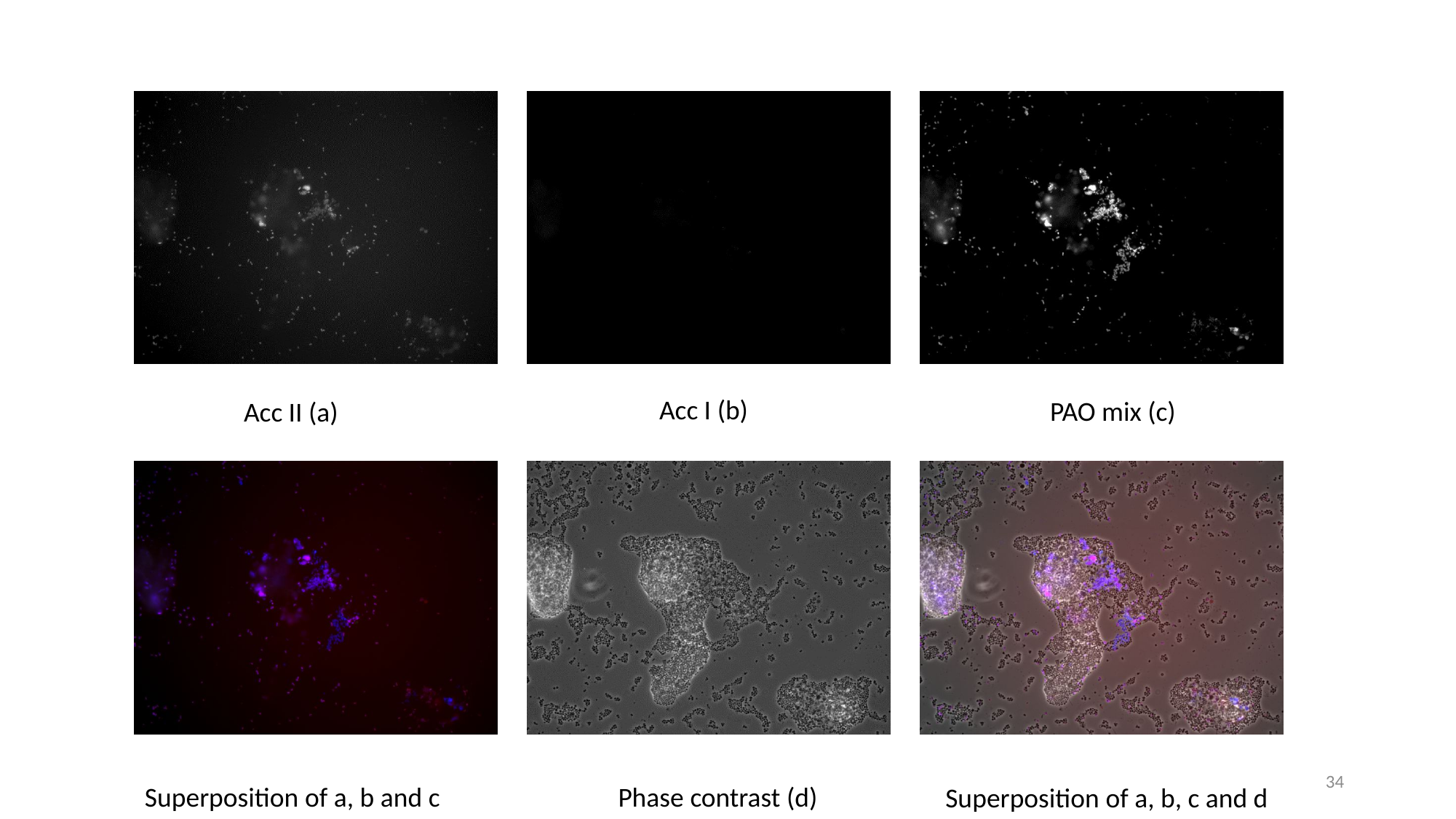

Acc I (b)
PAO mix (c)
Acc II (a)
34
Superposition of a, b and c
Phase contrast (d)
Superposition of a, b, c and d

## Slide 35
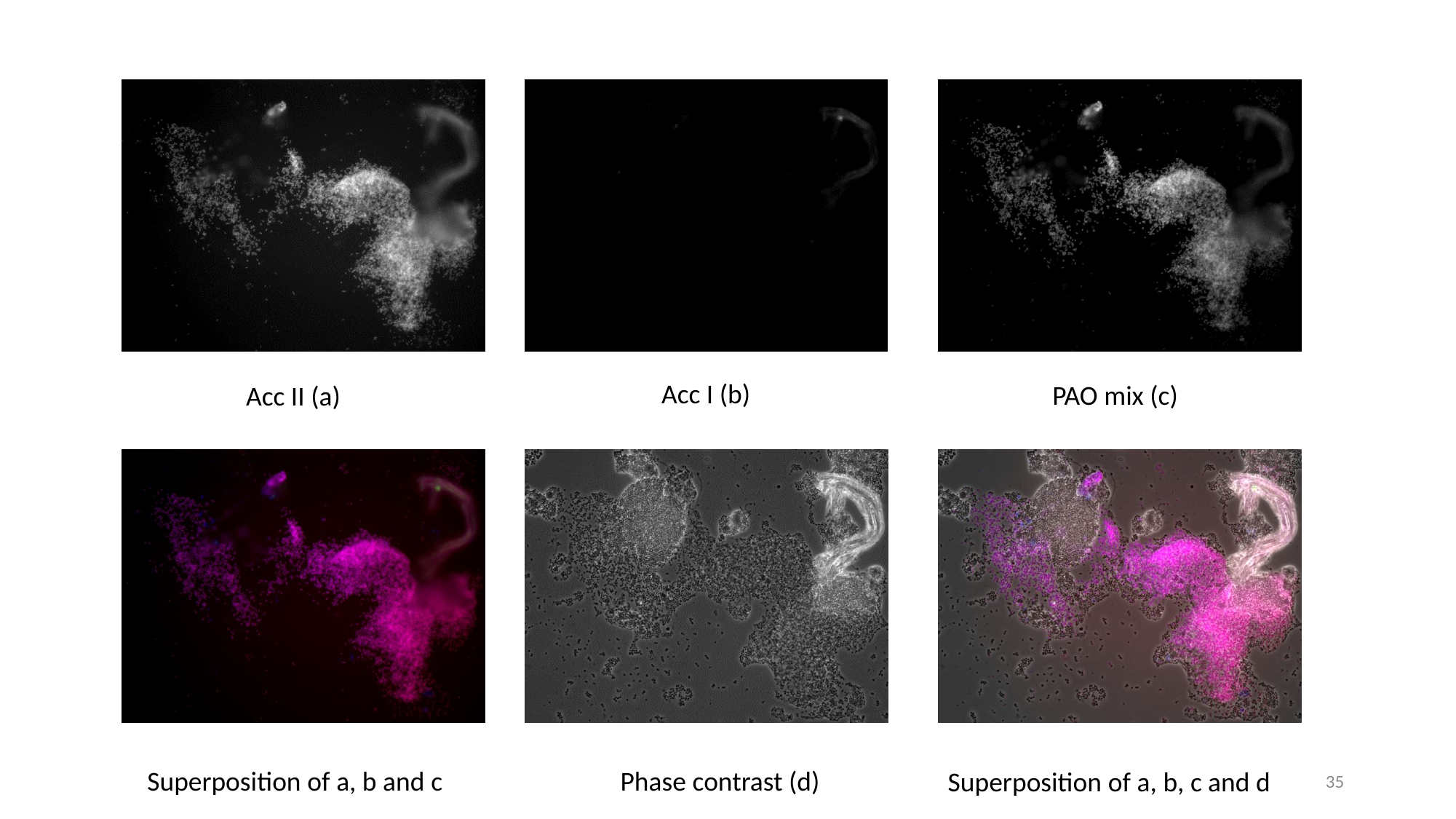

Acc I (b)
PAO mix (c)
Acc II (a)
Superposition of a, b and c
Phase contrast (d)
35
Superposition of a, b, c and d

## Slide 36
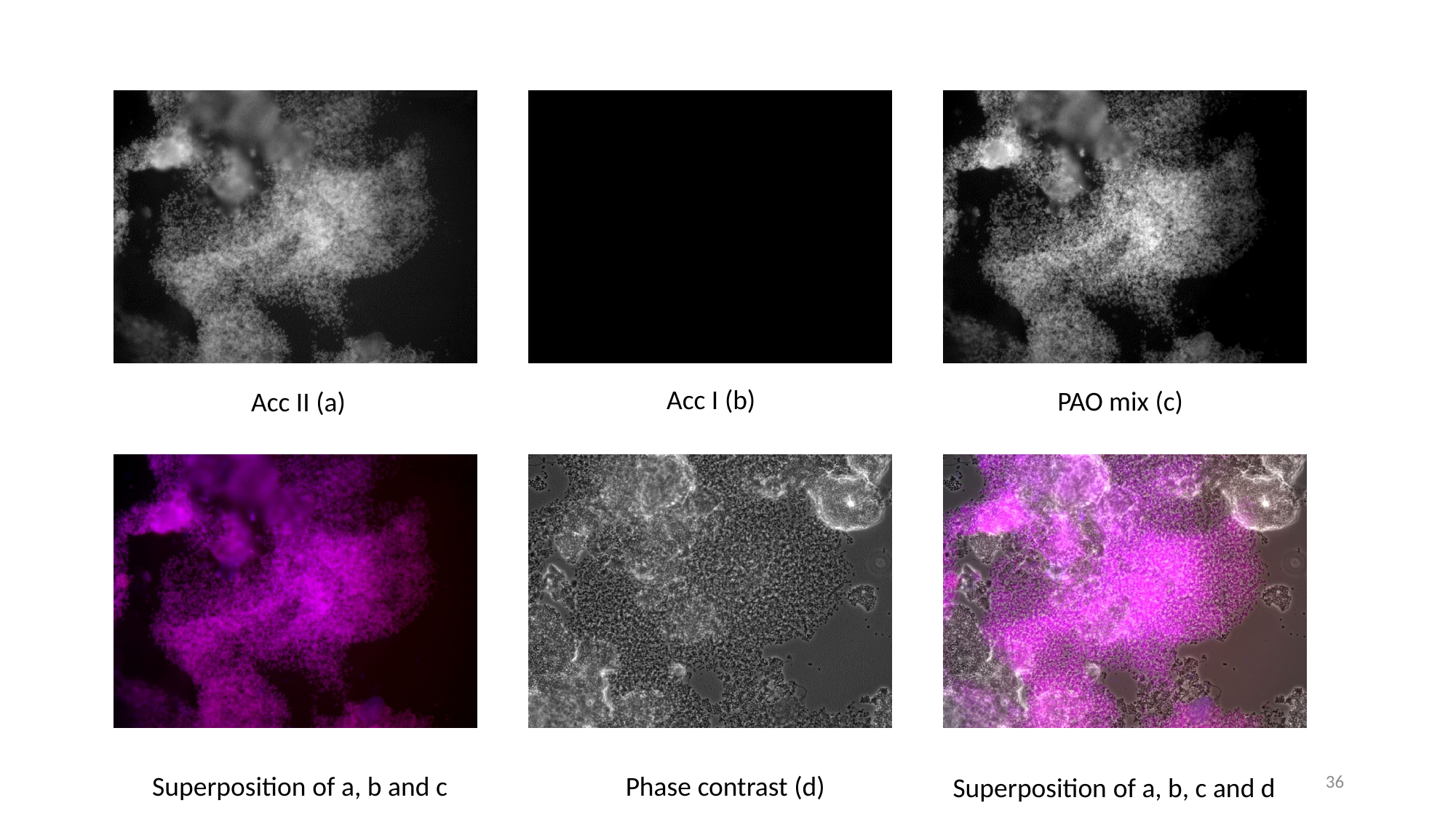

Acc I (b)
PAO mix (c)
Acc II (a)
36
Superposition of a, b and c
Phase contrast (d)
Superposition of a, b, c and d

## Slide 37
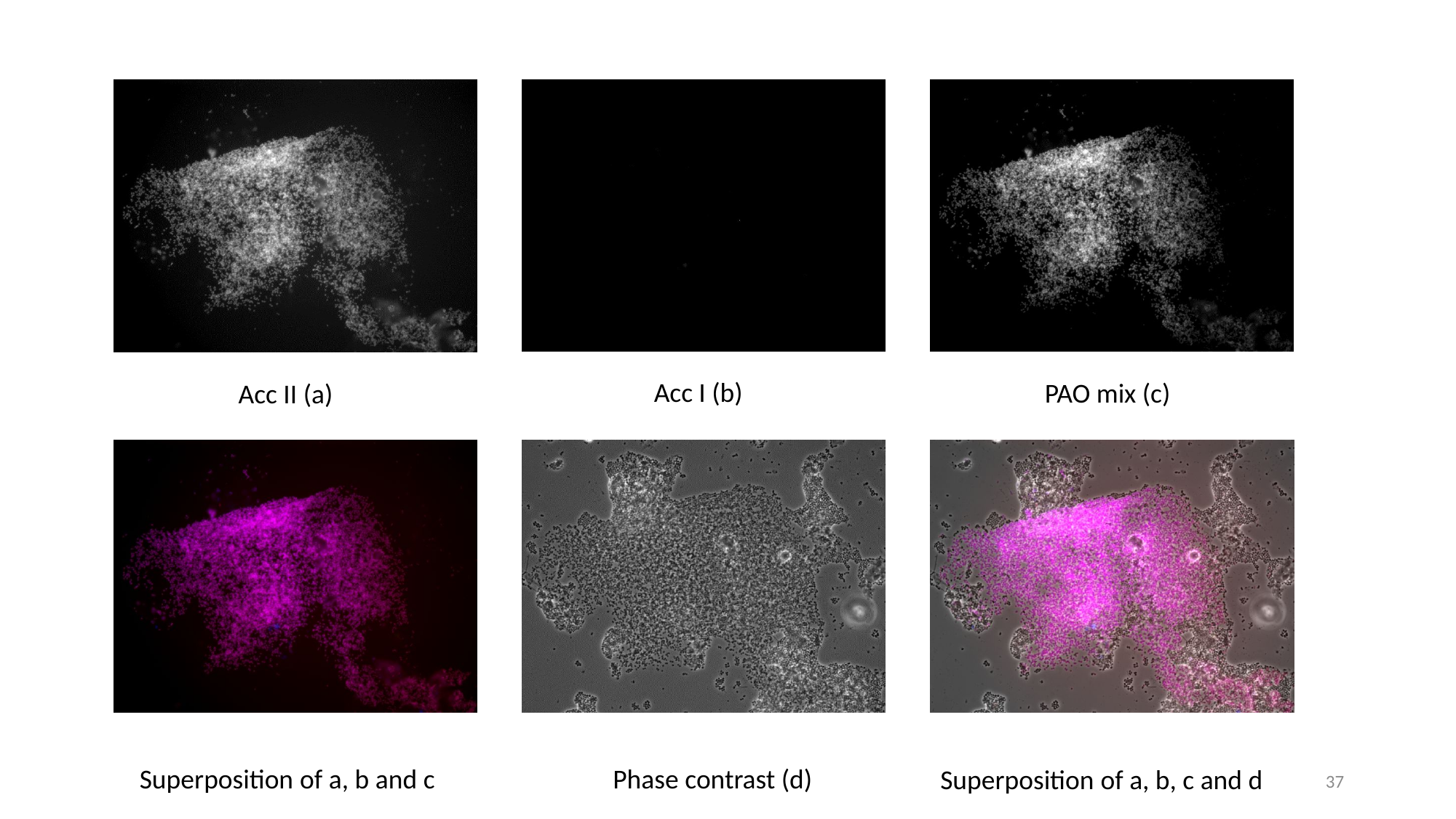

Acc I (b)
PAO mix (c)
Acc II (a)
Superposition of a, b and c
Phase contrast (d)
Superposition of a, b, c and d
37

## Slide 38
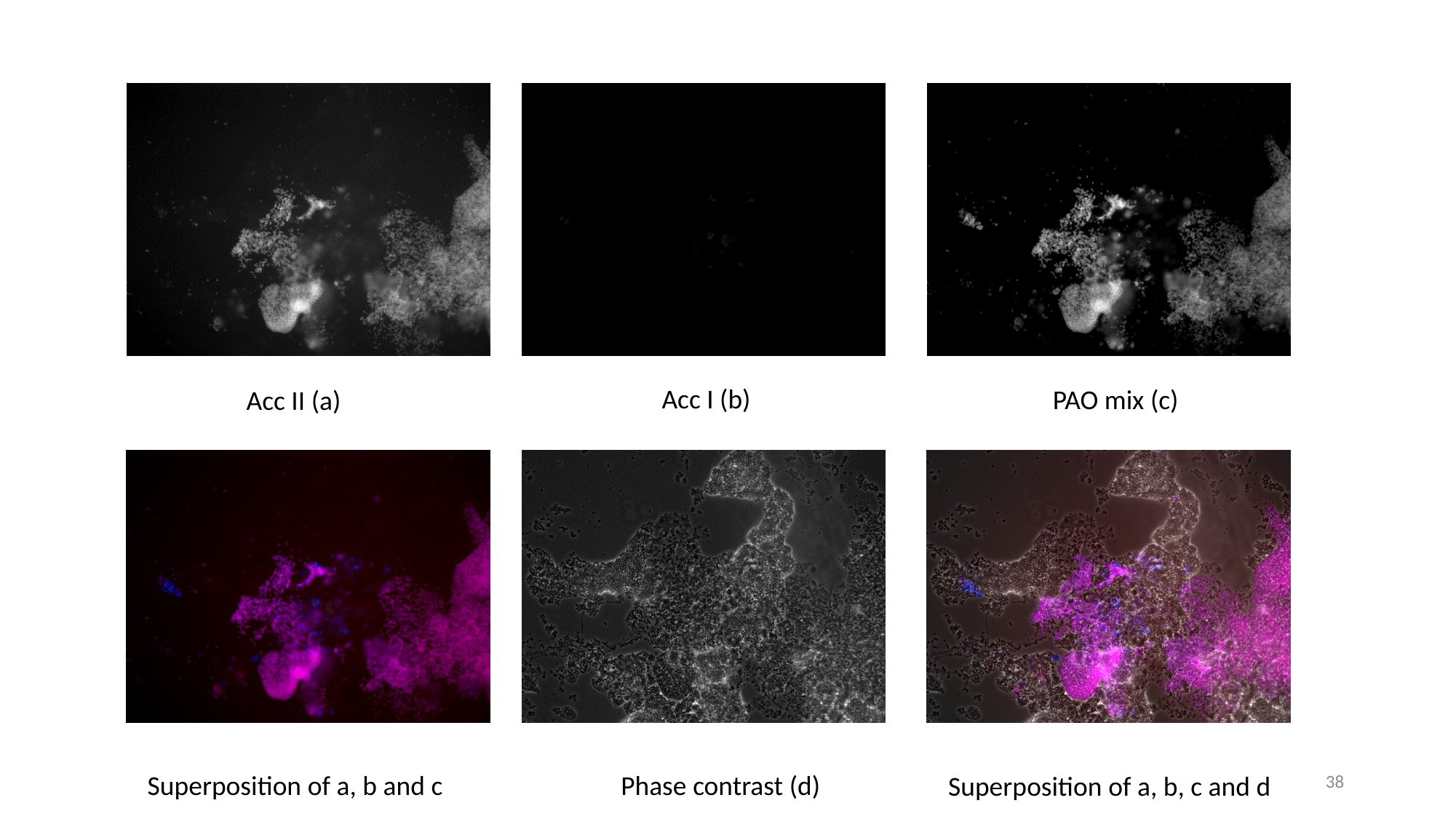

Acc I (b)
PAO mix (c)
Acc II (a)
38
Superposition of a, b and c
Phase contrast (d)
Superposition of a, b, c and d

## Slide 39
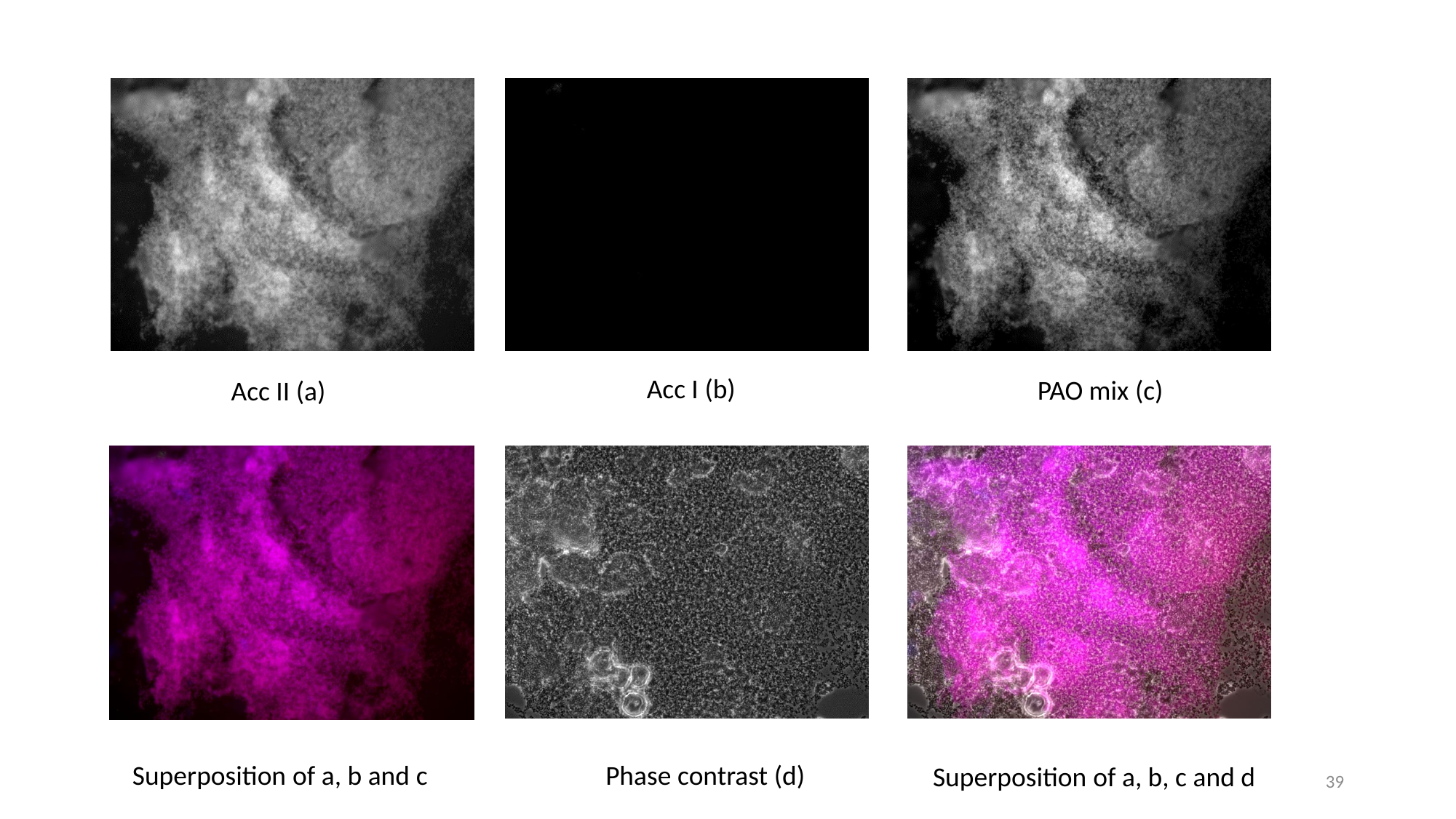

Acc I (b)
PAO mix (c)
Acc II (a)
Superposition of a, b and c
Phase contrast (d)
Superposition of a, b, c and d
39
